# Multi-Phenotype Association Decomposition: Unraveling Complex Gene-Phenotype Relationships

**DOI:** 10.3389/fgene.2019.00417

**Published:** 2019-05-10

**Authors:** Deborah Weighill, Piet Jones, Carissa Bleker, Priya Ranjan, Manesh Shah, Nan Zhao, Madhavi Martin, Stephen DiFazio, David Macaya-Sanz, Jeremy Schmutz, Avinash Sreedasyam, Timothy Tschaplinski, Gerald Tuskan, Daniel Jacobson

**Affiliations:** ^1^The Bredesen Center for Interdisciplinary Research and Graduate Education, University of Tennessee, Knoxville, TN, United States; ^2^Biosciences Division, Oak Ridge National Laboratory, Oak Ridge, TN, United States; ^3^Department of Plant Sciences, The University of Tennessee Institute of Agriculture, University of Tennessee, Knoxville, TN, United States; ^4^Department of Biology, West Virginia University, Morgantown, WV, United States; ^5^Department of Energy Joint Genome Institute, Walnut Creek, CA, United States; ^6^HudsonAlpha Institute for Biotechnology, Huntsville, AL, United States

**Keywords:** multi-phenotype associations, pleiotropy, GWAS, SNP clustering, networks, powerset space, pleiotropic signature, hypothesis generation

## Abstract

Various patterns of multi-phenotype associations (MPAs) exist in the results of Genome Wide Association Studies (GWAS) involving different topologies of single nucleotide polymorphism (SNP)-phenotype associations. These can provide interesting information about the different impacts of a gene on closely related phenotypes or disparate phenotypes (pleiotropy). In this work we present MPA Decomposition, a new network-based approach which decomposes the results of a multi-phenotype GWAS study into three bipartite networks, which, when used together, unravel the multi-phenotype signatures of genes on a genome-wide scale. The decomposition involves the construction of a phenotype powerset space, and subsequent mapping of genes into this new space. Clustering of genes in this powerset space groups genes based on their detailed MPA signatures. We show that this method allows us to find multiple different MPA and pleiotropic signatures within individual genes and to classify and cluster genes based on these SNP-phenotype association topologies. We demonstrate the use of this approach on a GWAS analysis of a large population of 882 *Populus trichocarpa* genotypes using untargeted metabolomics phenotypes. This method should prove invaluable in the interpretation of large GWAS datasets and aid in future synthetic biology efforts designed to optimize phenotypes of interest.

## 1. Introduction

Unraveling the complex genetic patterns underlying complex phenotypes has previously been challenging. While individual Genome-Wide Association Studies (GWAS) can provide insight into the genetic underpinnings of measured phenotypes, they typically involved associations of genetic variants with only one or a few phenotypes. The field of phenomics involves the collection of high-dimensional phenotype data of an organism, with the aim of capturing the overall, comprehensive phenotype (the “Phenome”) of the organism (Houle et al., 2010). Association studies involving many measured phenotypes, for example, Phenome-Wide Association Studies (PheWAS) present many advantages, in that they allow for the complex interconnected networks between phenotypes and their genetic underpinnings to be elucidated, and also allow for the detection of pleiotropy (Pendergrass et al., 2011, 2013, 2015; Hall et al., 2014).

**Figure 1:**
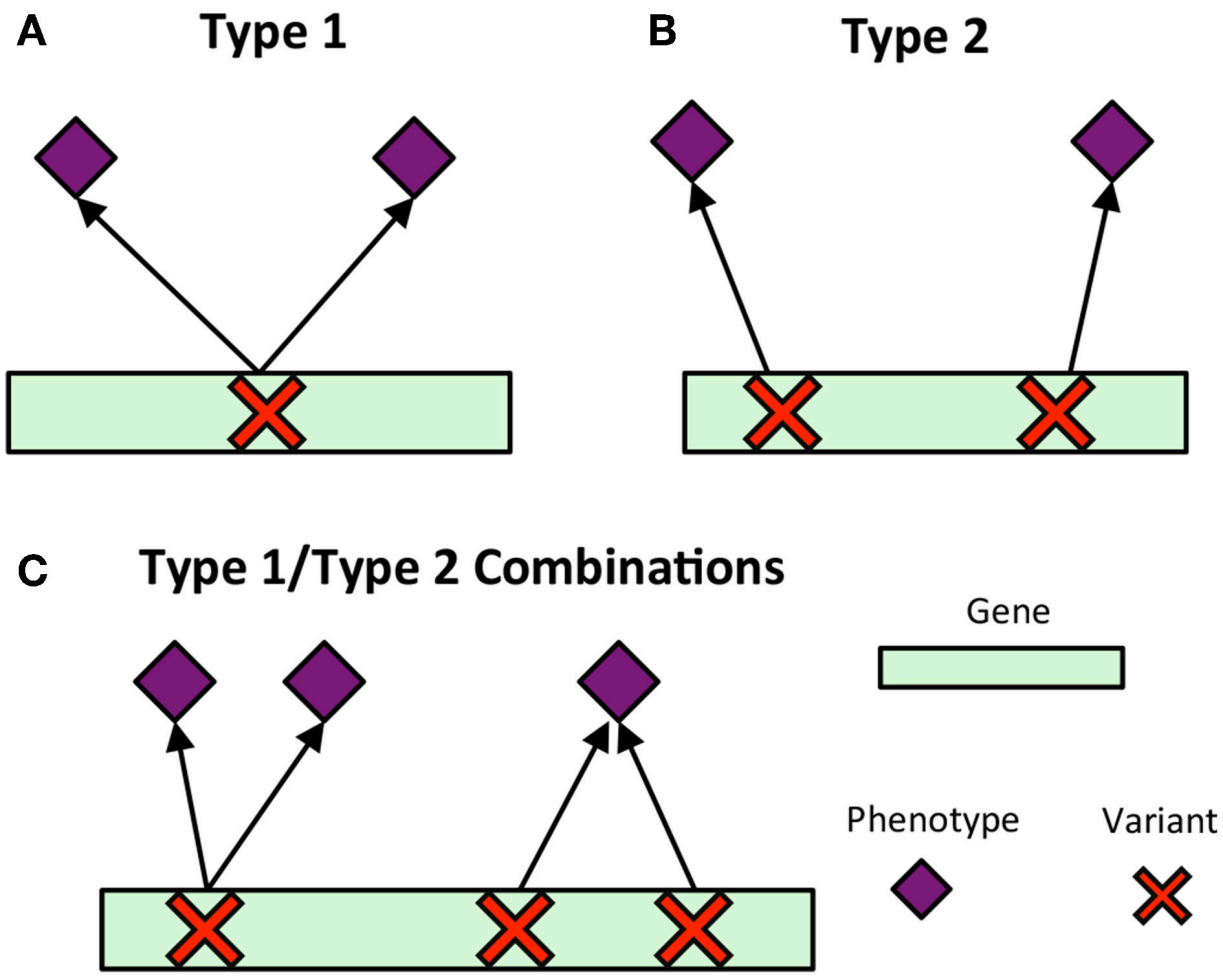
MPA signatures. **(A)** Type 1 MPA: a gene is associated with more than one phenotype due to a single variant within the gene associating with multiple phenotypes. **(B)** Type 2 MPA: a gene is associated with more than one phenotype because of alternate SNPs within the gene having different phenotypic associations (figure created from information presented in Solovieff et al., 2013). **(C)** Complex combinations of Type 1 and Type 2 signatures.

Pleiotropy is the phenomenon in which a gene affects multiple phenotypes (Tyler et al., 2009). One can also have a locus-centric view of pleiotropy involving a single SNP affecting multiple phenotypes (Solovieff et al., 2013). While pleiotropy used to be considered an exception to the rules of Mendelian genetics, it has since been proposed to be a common, central property inherent to biological systems (Tyler et al., 2009). Multi-phenotype associations (MPAs) can be detected in the results of Genome Wide Association Studies (GWASs) as Single Nucleotide Polymorphisms (SNPs) within genes/functional regions having multiple significant phenotype associations. This can be considered to be a pleiotropic pattern when the two phenotypes are seemingly unrelated. Two main MPA patterns exist within GWAS results. Type 1 MPAs occur when a single SNP within a functional region (such as a gene) is associated with more than one phenotype, whereas Type 2 MPAs occur when two different SNPs within a single functional region have different phenotype associations (Solovieff et al., 2013; Hackinger and Zeggini, 2017) (Figures 1A,B).

Multivariate analysis of the results of GWAS studies across many phenotypes have allowed for the investigation of complex relationships between genes and phenotypes, including pleiotropic relationships and the clustering of variants based on their phenotype associations. Many of these studies have involved the analysis of SNP associations with complex human disease traits. Some studies have considered pleiotropy as genes and SNPs associated with more than one phenotype, and found that pleiotropic genes tended to be longer, and that SNPs within pleiotropic genes were more likely to be exonic (Sivakumaran et al., 2011). Weighted Gene Co-expression Network Analysis (WGCNA) has been extended to cluster SNPs based on their phenotype associations using a matrix of beta coefficients, followed by hierarchical clustering of the Topological Overlap Matrix (Levine et al., 2017), and show how the resulting clusters can be used to produce polygenic scores. Gupta et al. (2011) introduced a biclustering algorithm, simultaneously clustering SNPs and phenotypes in a matrix of regression coefficients. Network-based approaches have been developed which construct bipartite networks of gene-disease phenotype associations from GWAS, and constructed network projections of this bipartite network resulting in disease similarity and gene-similarity networks (Goh and Choi, 2012). Though these studies provide a baseline of the use of multivariate and network approaches for the analysis of GWAS results, there is, to our knowledge, no method which characterizes detailed MPA signatures of genes and no method which clusters genes based on these detailed signatures. Simply clustering genes based on their phenotype associations will not capture the vast amount of combinatorial possibilities of type 1 and type 2 signatures any given gene can harbor (Figure 1C), especially when the multi-phenotype GWAS study involves millions of variants and hundreds of phenotypes.

Methods for multi-trait GWAS have also been developed, associating variants to groups of phenotypes (see for example Stephens, 2013; Furlotte and Eskin, 2015; Cichonska et al., 2016; Kaakinen et al., 2017a,b; Mägi et al., 2017; Porter and OReilly, 2017; Thoen et al., 2017). Mägi et al. (2017) and Kaakinen et al. (2017a) present interesting methods for identifying the association between SNPs/genes and multiple phenotypes by using the phenotypes as predictors in the modeling of the genotype. These are valuable methods for determining which phenotypes/sets of phenotypes a given gene or SNP is associated with that are more sophisticated than standard univariate GWAS approaches. These methods however do not focus on the ability to characterize and cluster genes based on the collection of topologies of SNP-phenotype associations within the gene.

We present MPA Decomposition and Signature Clustering, a network-based approach involving a constructed powerset space, in which clustering distinguishes between genes based on the detailed topology of their unique MPA signature. MPA decomposition is a post-GWAS/post-PheWAS approach with is designed to take the results of a multi-phenotype genome-wide association-type analysis (such as a standard, univariate GWAS run on several phenotypes or a multi-phenotype approach such as SCOPA (Mägi et al., 2017) and provides a framework allowing the precise mathematical representation of the architecture of variant-phenotype associations within regions (MPA/pleiotropic signatures), and thus allows these regions (such as genes) to be clustered based on these complex signatures.

## 2. Methods and Materials

### 2.1. Overview

**Figure 2:**
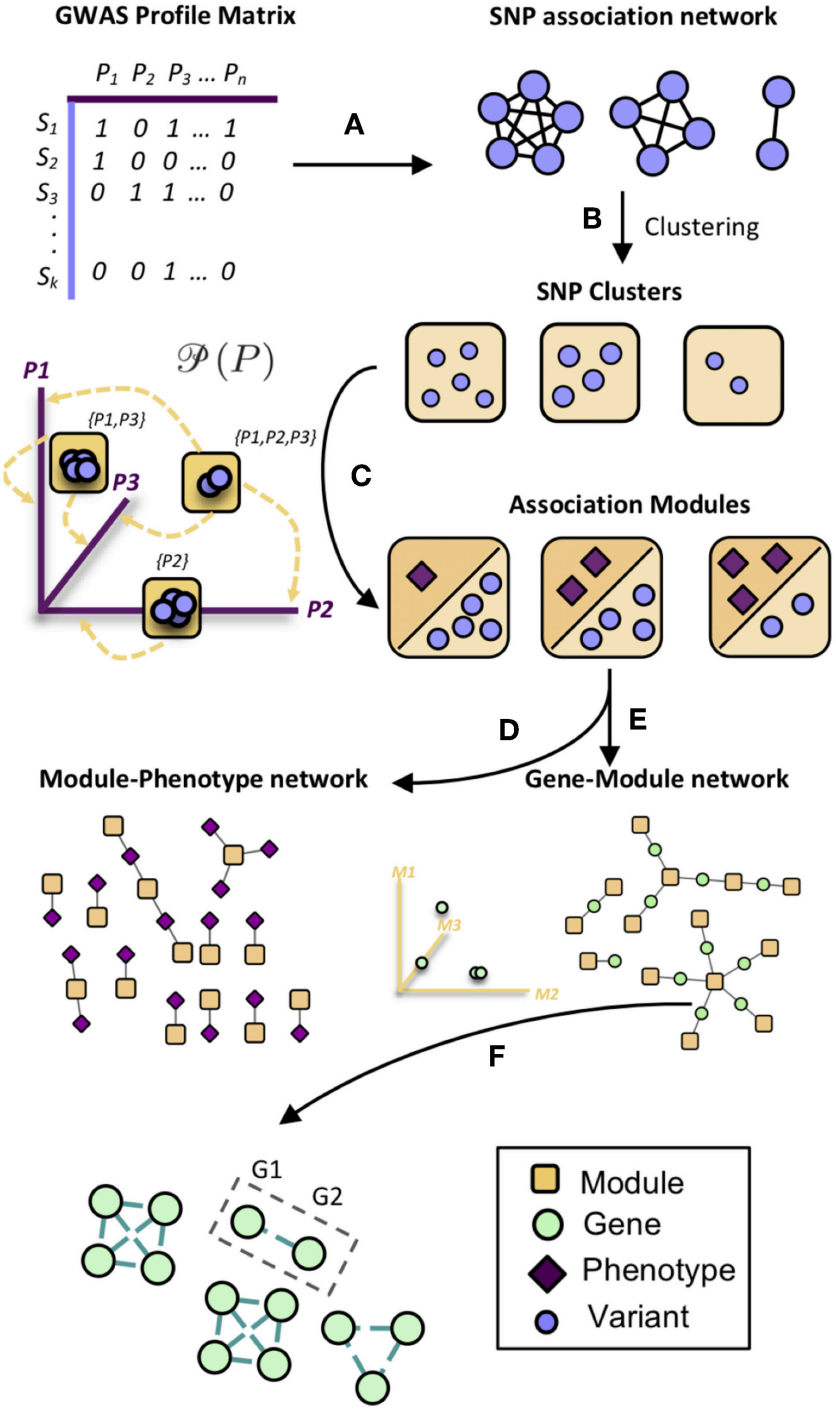
Overview of MPA decomposition and signature clustering. **(A)** The GWAS profile matrix *M* representing SNP-phenotype associations was constructed, and the Proportional Similarity between all pairs of SNPs (rows of *M*) was calculated. **(B)** Clustering of the SNP association similarity network results in clusters of SNPs with the same phenotype associations. **(C)** Association modules are constructed as elements of the powerset of phenotypes observed in the SNP clusters. Association modules can thus be seen as non-overlapping sets of SNPs, or overlapping sets of phenotypes. These modules form the axes of powerset space. **(D)** The module-phenotype network associates the phenotypes present in each element of the powerset observed in the SNP association clusters. **(E)** The gene-module network is constructed by mapping genes to association modules if the module contains a SNP that resides within that gene. **(F)** Signature clustering is performed in *GM* (powerset) space, grouping genes with the same module associations. Clustering genes in powerset space results in groups of genes with the same pattern of MPA signatures with the same set of phenotypes. For example, a signature cluster could involve G1 and G2 containing SNPs associating with both phenotypes P1 and P2, as well as a SNP associating with only P3.

MPA decomposition involves the mathematical characterization of each gene's MPA signature in a network-based context. This process begins in phenotype space. In this multi-dimensional space, each axis represents a phenotype and genes are represented as points, with points close together representing genes with similar phenotype associations and points far apart representing genes with very different phenotype associations. This phenotype space provides no information on the topology of associations within each gene. MPA decomposition maps genes to a newly constructed *powerset space*, which is constructed through clustering of SNP association vectors (Figures 2A–E). This clustering produces discrete sets of SNPs/overlapping sets of phenotypes called *association modules* which form the axes of powerset space, which provides the detailed structure of phenotype associations within a gene. The second stage—signature clustering—groups genes based on their detailed MPA signature (Figure 2F). Clustering of genes in this space results in groups of genes with identical MPA signatures. These genes grouped by MPA signatures provide a useful tool for the researcher planning genetic modification experiments, easily highlighting groups of genes with favorable signatures for modification to influence a particular phenotype.

The approach of MPA decomposition and its application are described below. MPA decomposition is a multi-step process whose results unify in a simple, matrix decomposition relationship. The multi-step process allows for the MPA signatures and signature clusters of genes to be determined from GWAS summary statistics, and is thus applicable to both newly generated genotype/phenotype data as well as published GWAS summary statistics. We apply and demonstrate this method on GWAS results from a densely genotyped *Populus trichocarpa* GWAS population involving approximately 10 million SNPs and over 400 untargetted metabolomics phenotypes measured across the population.

### 2.2. Metabolomics Genome-Wide Association Studies

Genotyping of 882 *P. trichocarpa* genotypes and metabolic profiling of 585 of these genotypes, followed by GWAS analysis of the 441 resulting metabolite phenotypes provided a network of associations between SNPs and metabolic phenotypes. The process for the construction of the GWAS network is described below.

#### 2.2.1. *Populus trichocarpa* SNPs

*P. trichocarpa* (Tuskan et al., 2006) SNP data (DOI 10.13139/OLCF/1411410) obtained from [https://doi.ccs.ornl.gov/ui/doi/55] was derived from the whole genome resequencing of a Genome Wide Association Study (GWAS) population clonally replicated in common gardens (Tuskan et al., 2011). This dataset consists of 28,342,758 SNPs called across 882 *P. trichocarpa* genotypes. Details on the generation of this SNP dataset can be found in Weighill et al. (2018). VCFtools (Danecek et al., 2011) was used to extract the most reliable set of SNPs corresponding to the 90% tranche, resulting in a set of 10,438,861 bi-allelic SNPs.

#### 2.2.2. Metabolomics Phenotypes

Untargetted metabolomics was conducted on *P. trichocarpa* genotypes using GC-MS. The metabolite analysis used is described in Tschaplinski et al. (2014). Briefly, samples were freeze dried for 48 h and then ground with a microWiley mill with a 20 mesh screen, with samples then twice extracted in 80% ethanol (aqueous) and the extracts combined before an aliquot was dried under nitrogen. Dried extracts were dissolved in acetonitrile followed by the addition N-methyl-N-trimethylsilyltrifluoroacetamide with 1% trimethylchlorosilane. Samples were heated for 1 h at 70°C to generate trimethylsilyl (TMS) derivatives. Samples were injected in an inert XL gas chromatograph-mass spectrometer (Agilent Technologies Inc., Santa Clara, CA, U.S.A.), fitted with an Rtx-5MS with Integra-Guard (5% diphenyl/95% dimethyl polysiloxane) capillary column (30 m by 250 μm by 0.25 μm film thickness) (Restek, Bellefonte, PA, U.S.A.). A standard quadrupole GC-MS was operated in the electron impact (70 eV) ionization mode, targeting 2.5 full-spectrum (50–650 Da) scans per second, as described previously (Tschaplinski et al., 2012). A large user-created database (>2,400 spectra) of mass spectral electron impact ionization fragmentation patterns of TMS-derivatized compounds, as well as the Wiley Registry 10th Edition with the NIST 2014 mass spectral database, were used to identify the metabolites of interest. Metabolites were quantified by extracting a key, characteristic mass-to-charge (m/z) for each known and unidentified metabolite using an automated data extraction program. Preprocessing of the resulting raw GC-MS data included alignment using XCMS (Smith et al., 2006) and normalization for amount of leaf sample analyzed, fraction of extracted sample analyzed, and internal standard recovered.

#### 2.2.3. Outlier Analysis

We performed outlier detection on each of the metabolomic phenotypes, to account for measurement variability and technical/experimental error, using R (R Core Team, 2013). This determines which, if any, metabolite intensities that are measured over the respective genotypes (individuals), are very different from the median observed intensities for that metabolite. We applied a variant of the method discussed in Leys et al. (2013), using the median absolute deviation (MAD) from the median. Our approach differs in that it takes into account the asymmetry of the distribution of intensity values, as lower intensities are more frequent. We thus calculated the MAD for the upper and lower tails of the distribution separately. By investigating the distribution of intensities and the MAD distance from the median, for a random sample of metabolites, we determined that a MAD distance of 5 is appropriate for outlier detection, this was done using the ggplot2 package in R (Wickham, 2009). Any intensity value of a metabolite for a given genotype that was more than 5 MADs from the median was removed from the analysis. Also, to mitigate potential biases from under-represented metabolites, we excluded any metabolite that had less than 100 non-zero, non-outlier values.

#### 2.2.4. GWAS

The EMMAX software (Kang et al., 2010) was used to statistically associate measured phenotypes with SNPs in *Populus trichocarpa*. Covariates were included to account for population structure by estimating a kinship matrix using the default parameters for Balding-Nichols method implemented in the emmax-kin program (Balding and Nichols, 1995). This was run in a parallel fashion using a customized Python script which made use of the NumPy (van der Walt et al., 2011), SciPY (http://www.scipy.org/) (Jones et al., 2001), pandas (McKinney, 2010) and mpi4py (Dalcín et al., 2005, 2008; Dalcin et al., 2011) modules. A hierarchical procedure similar to the approach described in Peterson et al. (2016), consisting of the Benjamini-Hochberg stepwise procedure (Benjamini and Hochberg, 1995) with a relaxed threshold of q1=0.1, together with the Gavrilov-Benjamini-Sarkar adaptive step-down procedure with a q2~7.9e-06, was applied to control the false discovery rate (FDR). Associations passing the respective thresholds were considered significant associations. A total of 413 phenotypes had at least one significant SNP association, and 131,282 SNPs had at least one significant phenotype association.

### 2.3. MPA Decomposition

The process for MPA decomposition described below is represented visually in Figure 2.

#### 2.3.1. GWAS Profile Matrix Construction

The GWAS profile matrix is the input to MPA decomposition (Figure 2). The GWAS profile matrix *M* was constructed in which each row represented a SNP that resides within a gene region, each column represented a phenotype and each entry *M*_*ij*_ was defined as:

(1) $$M_{ij}=\;\{\begin{array}{ll} 1 & \text{if SNP }i\text{ is associated with phenotype }j\\ 0 & \text{otherwise} \end{array}$$

Each row of the matrix *M* represents the GWAS profile of a particular SNP. SNPs were mapped to their respective genes using the *P. trichocarpa* version 3 genome annotation (Tuskan et al., 2006) available on Phytozome (Goodstein et al., 2012) through the genome portal of the Department of Energy Joint Genome Institute (Grigoriev et al., 2012; Nordberg et al., 2014). A gene was considered to consist of its coding sequences as well as regulatory elements such as 5′ and 3′ UTRs.

#### 2.3.2. Module Construction

The procedure for the construction of association modules is shown in Figure 2, steps A through C. The GWAS profiles of all pairs of SNPs in the GWAS profile matrix *M* were compared by calculating the Proportional Similarity Index between all pairs of rows of *M*. The Proportional Similarity Index between two vectors *X* and *Y* is defined as (Bloom, 1981):

(2) $$\begin{array}{l} PS\left(X,Y\right)=\frac{2\sum_{i}\min\left(x_{i},y_{i}\right)}{\sum_{i}\left(x_{i}+y_{i}\right)} \end{array}$$

where *X* and *Y* are the GWAS profiles of two SNPs (i.e., two rows of the matrix *M*), *x*_*i*_ is the *i*th entry in row *X* and *y*_*i*_ is the *i*th entry in row *Y*. This was performed in parallel using a customized Perl script which made use of the Parallel::MPI::Simple Perl module, developed by Alex Gough and available on The Comprehensive Perl Archive Network (CPAN) at www.cpan.org. This all-vs-all comparison results in a complete, unpruned SNP association network in which nodes represent SNPs and edges represent the similarity between the phenotype associations of SNPs.

We extracted association modules from the SNP association network as follows: First we identify SNPs that reside within genes with multiple phenotype associations (MPA genes). We extracted SNPs within MPA genes and the edges between these SNPs, and then pruned the network to only include edges between SNPs which have identical phenotype associations. This was achieved by applying a Proportional Similarity threshold of 1 (Supplementary Texts S1, S2). Nodes of the resulting subnetwork were then clustered into groups using MCL (Van Dongen, 2000, 2008) available from http://micans.org/mcl/. Each resulting cluster represents a group of SNPs with the same phenotype associations, i.e., a group of SNPs driven together by a particular set of phenotypes, or, an element of the powerset of phenotypes. These *modules* of phenotypes form the axes of the powerset space.

#### 2.3.3. Module-Phenotype (MP) Matrix Construction

The *MP* matrix was constructed by mapping modules to phenotypes which drive the association between SNPs within the module (Figure 2D). Thus, the *MP* matrix was constructed such that each entry *ij* was defined as 1 if phenotype *j* had a significant GWAS association with all SNPs in module *i*. This could alternatively be seen as creating a network by connecting phenotype nodes to module nodes if that phenotype has a GWAS association with all SNPs in that module.

#### 2.3.4. Gene-Module (GM) Matrix Construction

The *GM* matrix was constructed by mapping modules to genes which contained SNPs within that module (Figure 2E). Thus, the *GM* matrix was constructed such that each entry *ij* was defined as 1 if module *j* contained a SNP that resides within gene *i*, and zero otherwise. This can also be seen as constructing a network by connecting gene nodes to module nodes which contain SNPs that reside within that gene region.

#### 2.3.5. Signature Clustering

Signature clustering (Figure 2F) was performed by calculating the similarity between all pairs of rows (genes) of the *GM* matrix using the proportional similarity metric, applying a threshold of 1, and clustering the resulting similarity network using MCL (Van Dongen, 2000, 2008).

### 2.4. Annotation and Functional Enrichment

*P. trichocarpa* gene boundaries as defined in the Ptrichocarpa_210_v3.0.gene.gff3 annotation file obtained from version 3 genome annotation (Tuskan et al., 2006) available on Phytozome was used. Functional annotations of *P. trichocarpa* genes were obtained from version 3 genome annotation (Tuskan et al., 2006) available on phytozome (Goodstein et al., 2012) through the genome portal of the Department of Energy Joint Genome Institute (Grigoriev et al., 2012; Nordberg et al., 2014).

Mapman annotations of *P. trichocarpa* were obtained by splitting the protein translations of *P. trichocarpa* genes into three sets and using the Meractor tool (Lohse et al., 2014) to assign Mapman terms to each gene. The BINGO Cytoscape plugin Maere et al. (2005) was used to determine enriched Gene Ontology (GO) terms in the set of type 1 and type 2 MPA genes.

### 2.5. Co-expression Network

A *P. trichocarpa* gene co-expression network was constructed as described in Weighill et al. (2018) making use of the *P. trichocarpa* (Nisqually-1) RNA-seq data derived from JGI Plant Gene Atlas project (Sreedasyam et al., unpublished), consisting of samples for various tissues (leaf, stem, root and bud tissue) and libraries generated from nitrogen source study. A list of sample descriptions was accessed from Phytozome at https://phytozome.jgi.doe.gov/phytomine/aspect.do?name=Expression.

## 3. Results and Discussion

### 3.1. MPA Decomposition: Construction of a New Space

**Figure 3:**
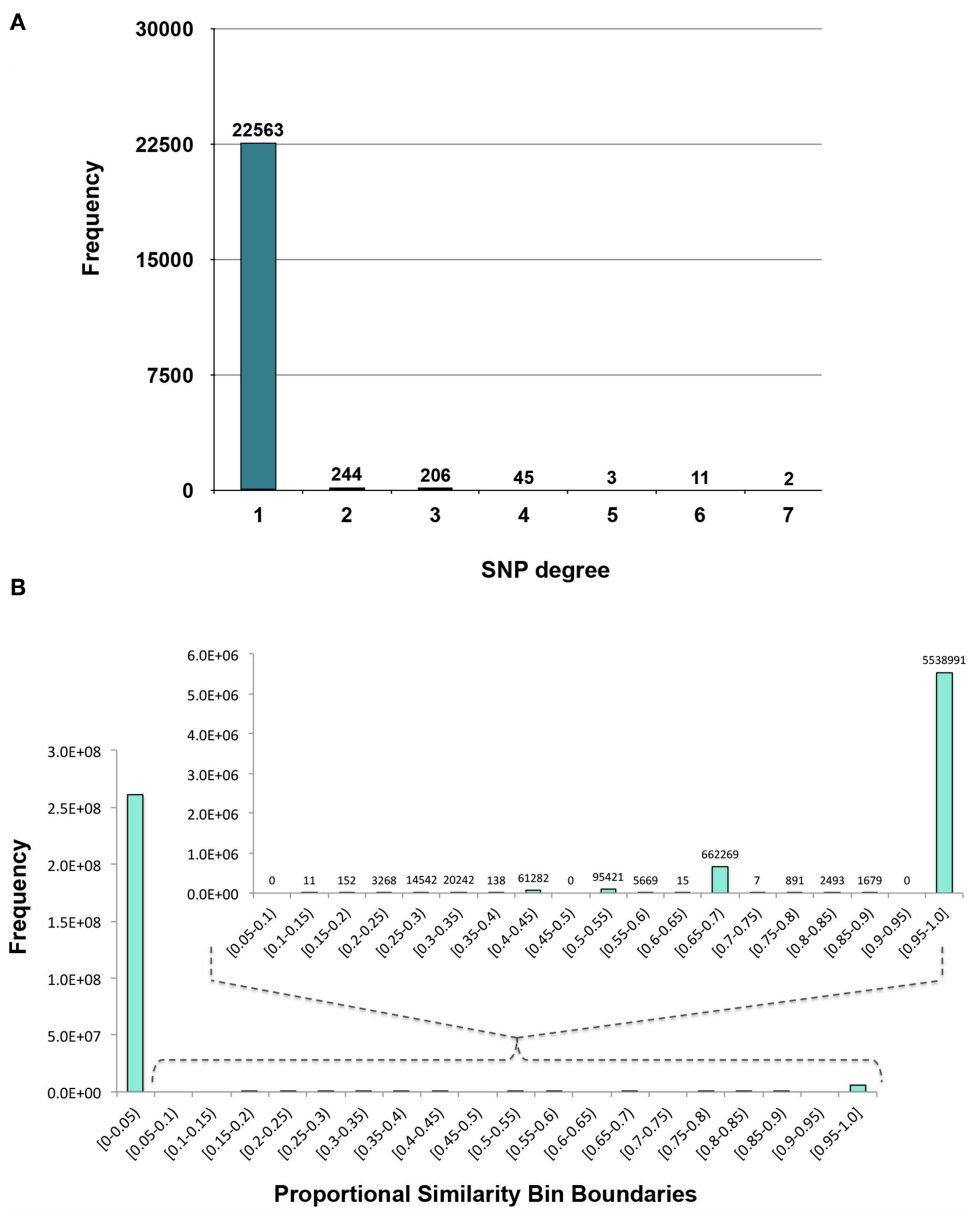
Distributions. **(A)** Degree distribution of SNP nodes in the SNP-phenotype GWAS bipartite network. **(B)** Distribution of the Proportional Similarity edge weights in the SNP association network.

**Figure 4:**
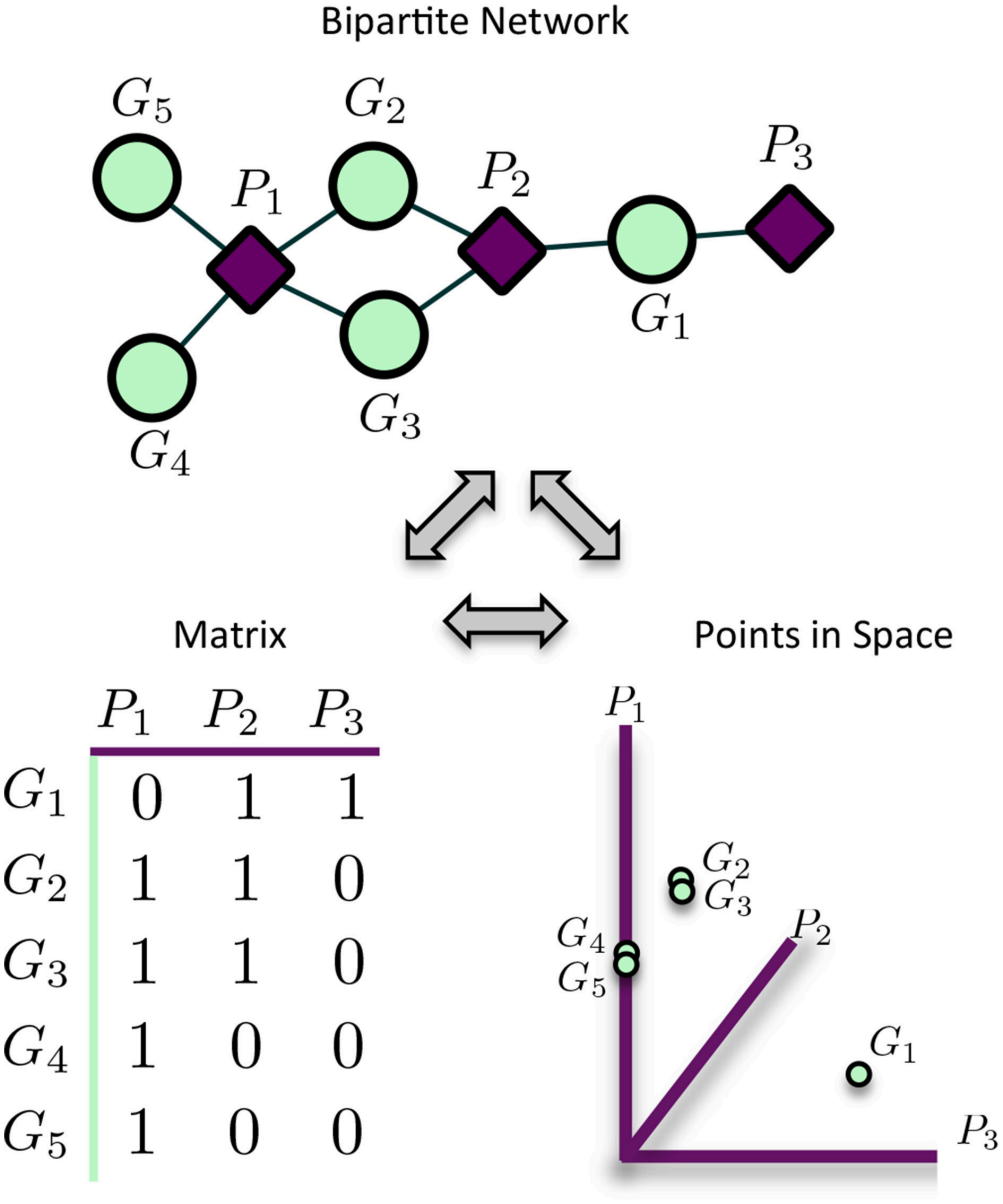
Representation of matrices as spaces and bipartite networks. Matrices of GWAS results can easily be represented as points in high dimensional space, with rows representing points and columns representing variables/axes. Equivalently, matrices can be represented as bipartite networks, connecting row objects (genes) with column variables if the corresponding entry is non-zero. This provides a useful way to visualize high dimensional spaces as bipartite networks.

MPA decomposition is a multi-step process which involves the construction of a new space, allowing for the multi-phenotype signatures of genes to be easily interpreted and clustered. This method makes use of bipartite networks as data structures. Bipartite networks represent connections (edges) between two classes of objects (nodes). The results of a standard GWAS analysis were represented as a bipartite SNP-phenotype network, connecting SNP nodes to phenotype nodes between which there were significant associations. While most SNPs had only a single phenotype association, there were several SNPs which had significant associations with multiple metabolite phenotypes (Figure 3A). Mapping SNPs from the GWAS associations to the genes in which they reside resulted in gene-phenotype associations, which can be represented as multiple different data structures. Firstly, genes can be represented as points in multi-dimensional phenotype space, indicating their respective phenotype associations (Figure 4). The closer genes are to each other in phenotype space, the more shared phenotype associations they have. Alternatively, these associations can be represented as a gene-phenotype (*GP*) bipartite network, linking a gene *g*_*i*_ to phenotype *p*_*k*_ if *g*_*i*_ contained a SNP significantly associated with *p*_*k*_ (Figure 4). Bipartite networks are useful for the visualization and investigation of points in high dimensional space, as well as for the representation of complex relationships between multiple objects. Thus, bipartite networks were used throughout MPA decomposition as the mathematical foundation as well as a visualization tool.

**Figure 5:**
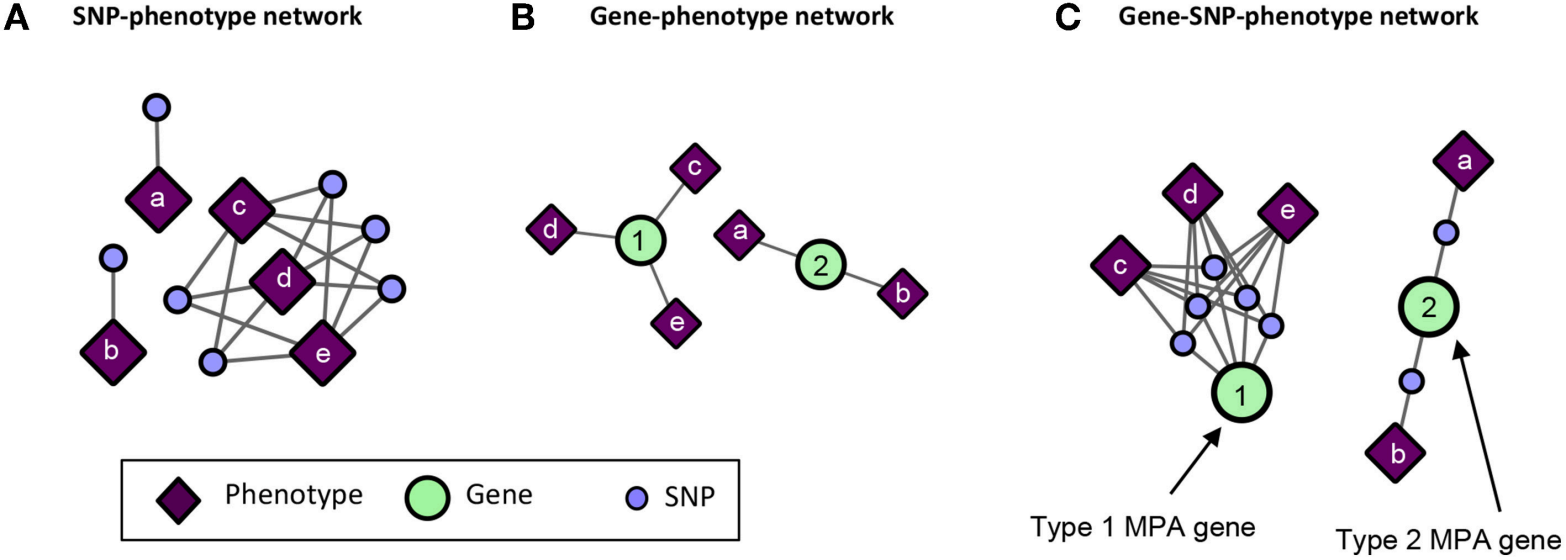
Example of SNP-phenotype, gene-phenotype networks and gene-SNP-phenotype networks. **(A)** SNP-phenotype bipartite networks simply connect SNPs to phenotypes with which they have a significant association, and do not provide information regarding MPA signatures within genes. **(B)** Gene-phenotype networks contain connections between genes and phenotypes. An edge will be drawn between a gene and a phenotype if that gene contains a SNP associated with that phenotype. Gene-phenotype networks do not provide information as to which type of MPA signature is exhibited. **(C)** Gene-SNP-phenotype networks are SNP-phenotype networks with the SNPs connected to genes in which they reside. These networks are more complicated, and MPA signatures can be deduced from their structure through further analysis, however, the network is not in a form in which MPA signatures can be extracted easily using standard network topology measures such as degree.

GWAS associations represented as a bipartite network of SNPs connected to their associated phenotypes (Figure 5A) do not give any indication of MPA signatures as there is no obvious information about which SNPs belong to which genes. Thus, bipartite SNP-phenotype networks give no indication of how many phenotype associations a given gene has. GWAS associations represented as a bipartite network of genes connected to their associated phenotypes (Figure 5B) can give an indication as to whether or not a gene has multiple phenotype associations in that it is associated with more than one phenotype, but cannot give any indication as to the type of MPA signature (type 1 or type 2) exhibited by the gene. Mapping the SNPs in the SNP-phenotype network to the genes in which they are present results in a gene-SNP-phenotype network (Figure 5C). From this network, it is possible to deduce the type of MPA signature exhibited by a gene through some amount of visual inspection, for example, looking at the SNPs within a gene and what their associated phenotypes are. However, the structure of this network does not allow the MPA signature of a gene to be readily extracted using simple node properties such as degree. For example, one cannot simply calculate the connectivity (degree) of each gene node in Figure 5C in order to determine the type of MPA signature exhibited, since one can have multiple SNPs within the same gene associating with the same set of phenotypes. In addition, it is not easy to determine which genes exhibit the same MPA signatures. The process of MPA decomposition allows one to maintain the topology of SNP associations within a gene while still being able to determine the type of MPA signature using simple network measures such as degree.

The first phase of MPA decomposition involved the construction of module space, a new multi-dimensional space in which each dimension/axis represented a particular subset of phenotypes. The powerset of a set is the collection of all possible subsets of that set. Thus, we can refer to the module space as “powerset space,” as each axis of the space is defined by a particular subset of phenotypes which are observed as co-associating phenotypes in the GWAS results. Modules of SNPs with the same co-associating phenotypes were identified using the Proportional Similarity metric. The distribution of Proportional Similarity values can be seen in Figure 3B. Of the pairs of SNPs which have non-zero Proportional Similarity values (i.e., those pairs of SNPs which shared at least one phenotype association), many had a proportional similarity value of 1. This is explained by the degree distributions of the SNPs in the original SNP-phenotype GWAS network (Figure 3A). The degree distribution of a network indicates the probability (or, in this case, frequency) at which a node can be found to have a certain number of edges connected to it (Barabási and Oltvai, 2004). Therefore, the distribution in Figure 3A indicates that, of the SNPs which had significant phenotype associations, most of them had precisely one phenotype association. This could skew the Proportional Similarity distribution since any pairs of these “1-phenotype-hit” SNPs which are associated with the same phenotype will have a Proportional Similarity index of 1. However, it is important to keep in mind that these “1-phenotype-hit” SNPs can still contribute to MPA signatures within genes, as two “1-phenotype-hit” SNPs within the same gene that have different associations is precisely what we define as Type 2 MPA signatures.

The modules form the building blocks of MPA signatures, and also conveniently collapse SNPs that are close together in genes and associate with the same set of phenotypes, and thus likely in LD. While representing non-overlapping sets of SNPs, these modules also represented overlapping sets of phenotypes. In particular, each module represented the set of phenotypes which were associated with all SNPs within the module. Thus, each module also represented an element of the powerset of phenotypes *P*(*P*) observed in the SNP-phenotype GWAS associations. These observed elements of the powerset were used to construct the powerset space, with each element/module representing a different dimension of this space.

**Figure 6:**
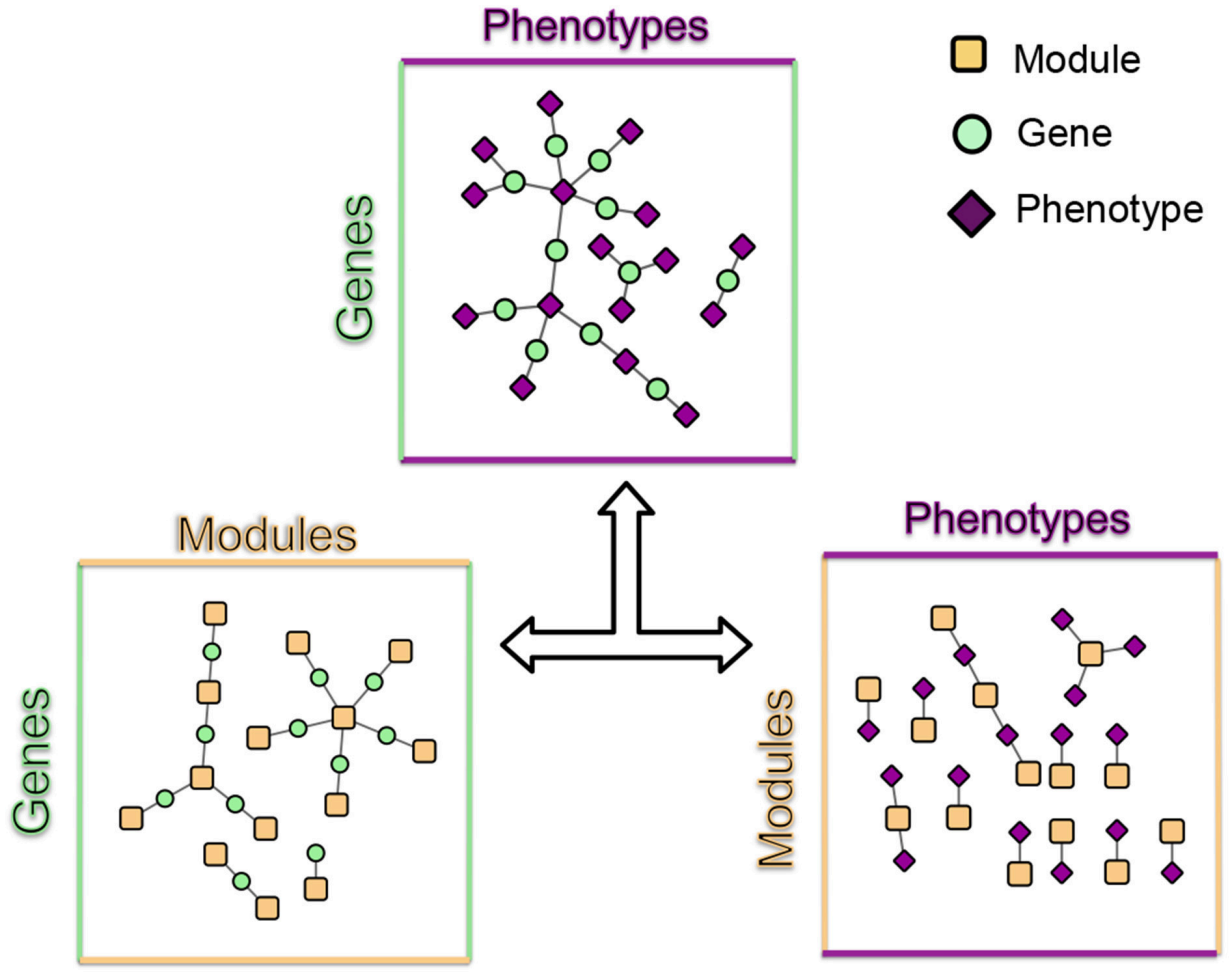
MPA decomposition. The gene-phenotype matrix is decomposed into two matrices, a gene-module (*GM*) matrix and a module-phenotype (*MP*) matrix (Supplementary Texts S3, S4, Supplementary Figure 1). The *GM* matrix represents genes in powerset space. *Association modules* (elements of the powerset of phenotypes) form the basic units of MPAs and are considered latent variables. Signature clustering is performed on genes in module space (*GM* matrix).

**Figure 7:**
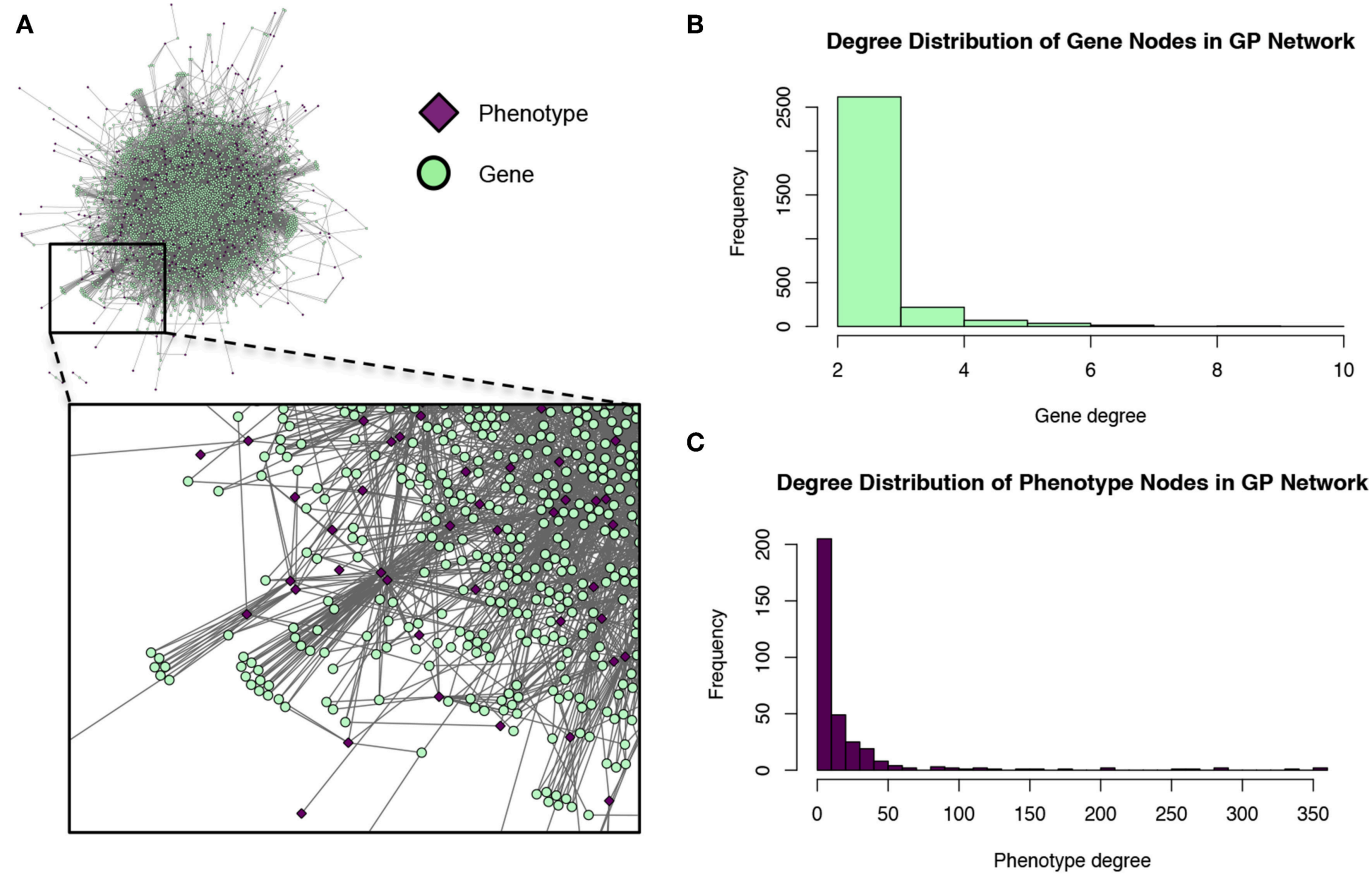
Gene-phenotype (*GP*) network. **(A)** The *GP* network. Green nodes represent MPA genes, pink diamonds represent metabolites (phenotypes). An edge connects a gene to a phenotype if that gene contains a SNP associated with that phenotype. **(B)** Degree distribution of the gene (green) nodes in the *GP* network. **(C)** Degree distribution of the phenotype (pink) nodes in the *GP* network.

**Figure 8:**
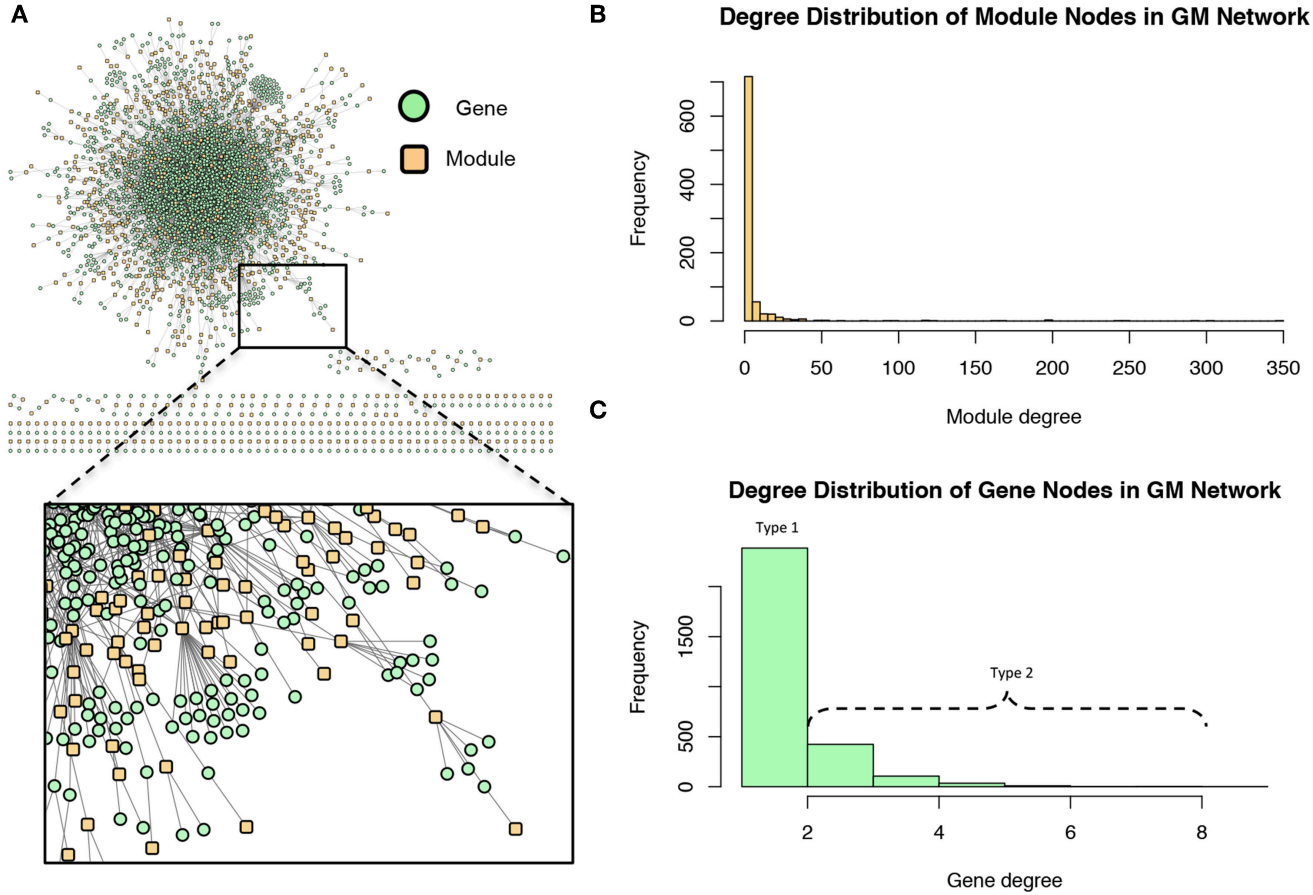
Gene-module (*GM*) network. **(A)** The *GM* network. Green nodes represent MPA genes and yellow nodes represent association modules. A gene node is connected to a module node if the module contains a SNP which resides within that gene. **(B)** Degree distribution of the module (yellow) nodes in the *GM* network. **(C)** Degree distribution of the gene (green) nodes in the *GM* network.

**Figure 9:**
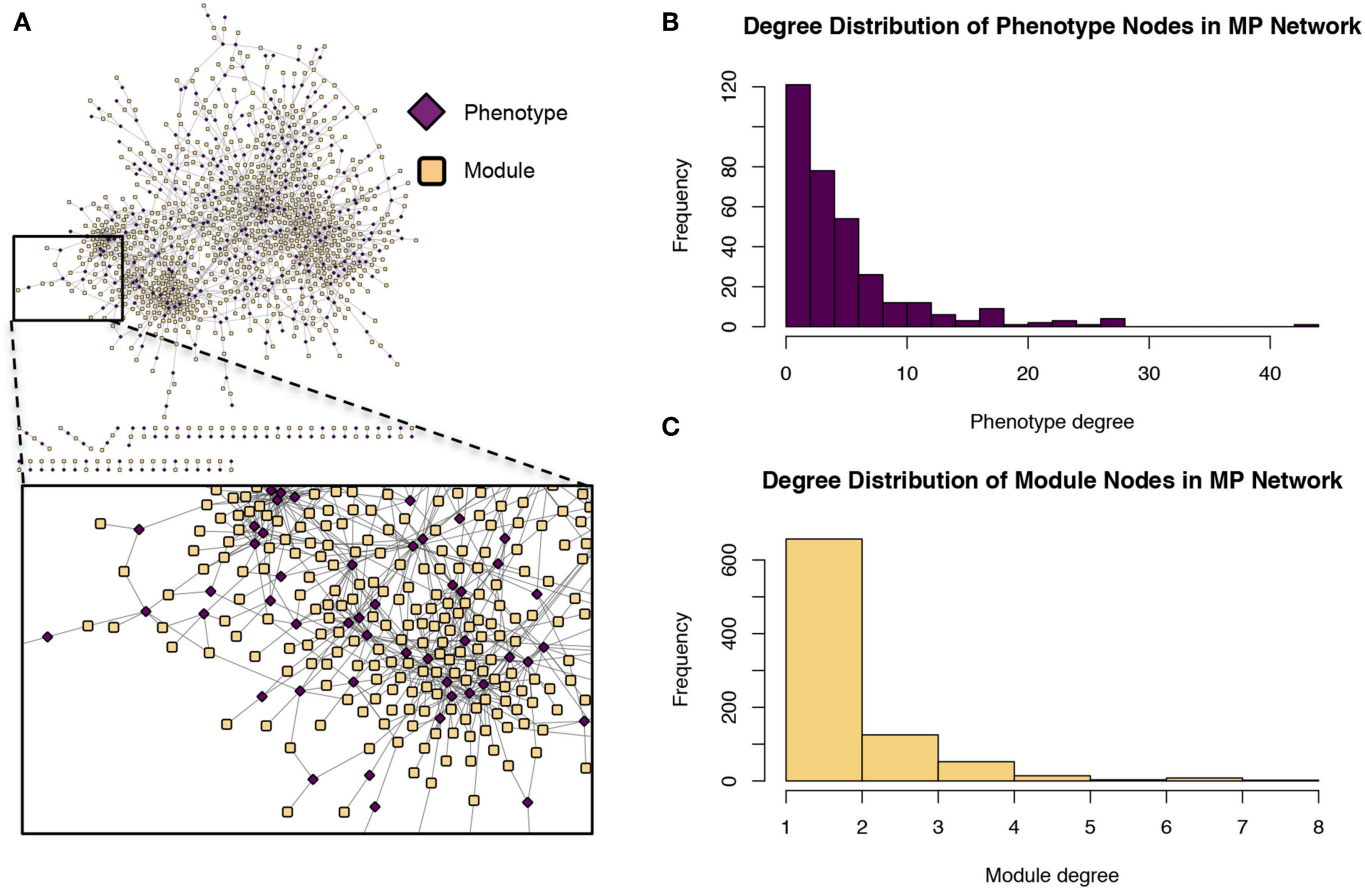
Module-phenotype (*MP*) network. **(A)** The *MP* network. Yellow nodes represent association modules and pink nodes represent phenotypes. A module node is connected to a phenotype node if the phenotype is associated with all SNPs within the module and is thus considered a driving phenotype of the module. **(B)** Degree distribution of the phenotype (pink) nodes in the *MP* network. **(C)** Degree distribution of the module (yellow) nodes in the *MP* network.

These modules allowed for the construction of the gene-module (*GM*) and the module-phenotype (*MP*) matrices, which are referred to as the decomposition matrices. Represented as bipartite networks, the *MP* bipartite network defined the axes of powerset space, and the *GM* bipartite network mapped the genes into powerset space. While phenotype space provided information as to the individual phenotype associations of genes, powerset space indicated a gene's associations with sets of phenotypes at the SNP level, providing a detailed MPA signature. The mapping from phenotype space to powerset space results in a decomposition relationship between the *GP*, *GM* and *MP* matrices (Figure 6, Supplementary Texts S3–S5, Supplementary Figure 1). In the *GP* network (Figure 7), nodes represented either genes or phenotypes, and an edge was defined between gene *G*_*i*_ and phenotype *P*_*j*_ if gene *G*_*i*_ contained a SNP which was statistically associated with phenotype *P*_*j*_ in the GWAS analysis. Nodes in the *GM* network (Figure 8) represented either genes or modules, and an edge was defined between gene *G*_*i*_ and module *M*_*j*_ if *M*_*j*_ contained a SNP that resided within gene *G*_*i*_. Nodes in the *MP* network (Figure 9) represented either association modules or phenotypes, and an edge was defined between module *M*_*i*_ and phenotype *P*_*j*_ if the correlation of SNPs within *M*_*i*_ is driven by phenotype *P*_*j*_.

### 3.2. Powerset Space Unravels Multi-Phenotype Association Signatures

The *GP* network (Figure 7) represents genes in phenotype space, and provides information regarding which genes are associated with which phenotypes, and can thus indicate which genes have multiple phenotype associations and are potentially pleiotropic. Of the 41,335 genes in *P. trichocarpa*, 2,964 genes had GWAS hits with more than 1 metabolite phenotype each, and are thus considered MPA genes with respect to the metabolic phenotypes.

**Figure 10:**
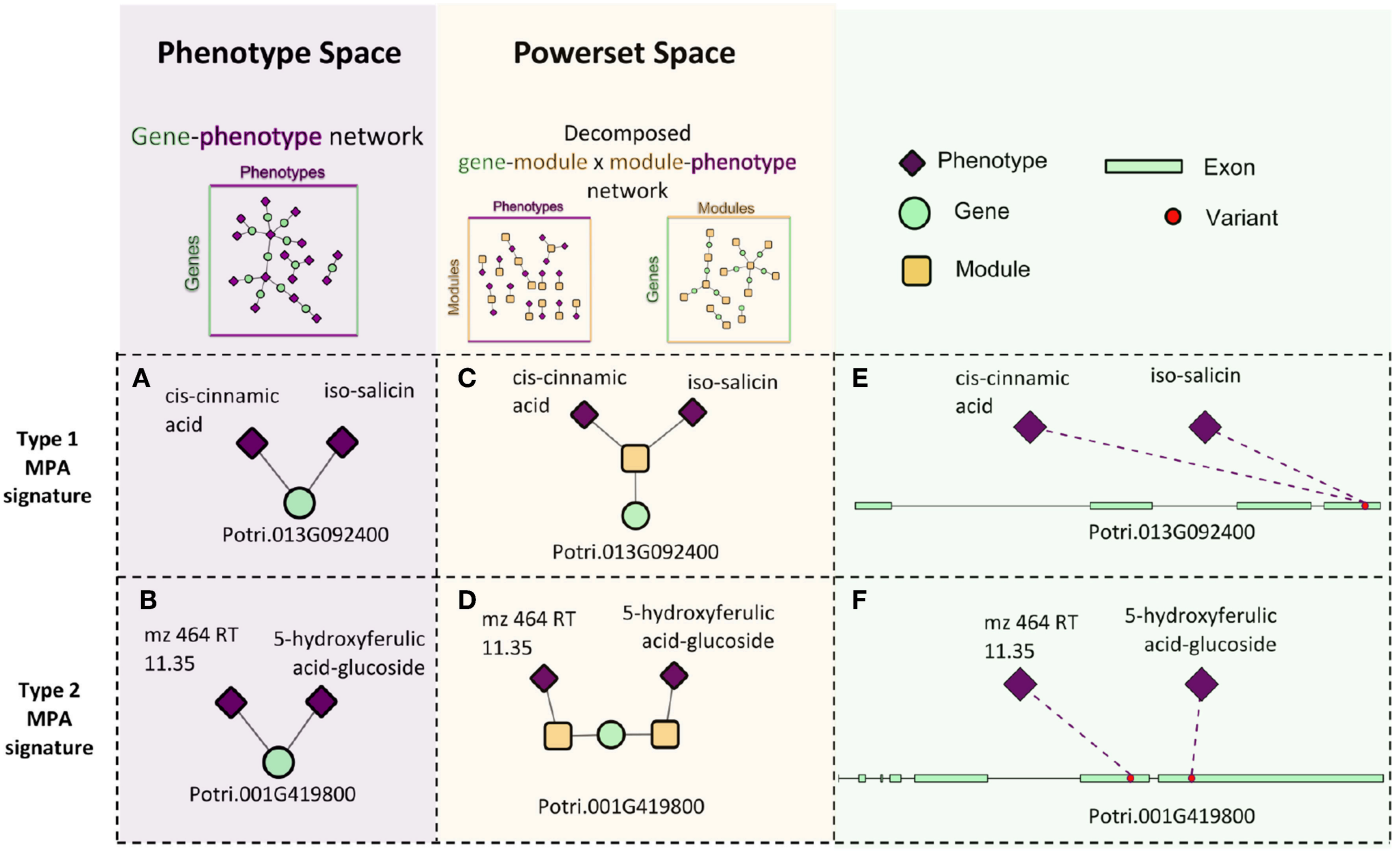
Signature decomposition example. Two genes, Potri.013G092400 **(A)** and Potri.001G419800 **(B)** have the same surrounding network topology in the *GP* network in that they are both connected to two phenotypes. Projecting the genes into powerset space through MPA decomposition of the *GP* network indicates that they exhibit different MPA signatures in that Potri.013G092400 exhibits a type 1 MPA signature **(C)**, containing a SNP associating with two phenotypes **(E)** and Potri.001G419800 exhibits a type 2 MPA signature. **(D)** containing two SNPs, each with a different phenotype association **(F)**.

The *GM* network (Figure 8) represents genes in powerset space, which in turn is defined by the *MP* network (Figure 9). The *GM* network unravels the MPA signatures of genes, representing their associations with sets of phenotypes. Genes that are connected to one module exhibit a Type 1 MPA signature because they contain SNPs which are associating with the same set of phenotypes, whereas genes connected to more than one module exhibit a Type 2 MPA signature because they contain SNPs which associate with different sets of phenotypes. Mapping of genes to module space thus reveals the Type 1 and Type 2 MPA patterns, as well as complex combinations of Type 1/Type 2 patterns that exist within genes (Figure 10). Phenotype associations of genes cannot be distinguished as Type 1 or Type 2 in phenotype space, whereas module space clearly indicates the MPA signature exhibited by a gene (Figure 10). Module space also goes beyond classifying genes as exhibiting Type 1 or Type 2 MPA signatures, but characterizes each unique topology of variant-phenotype associations within a gene separately. Thus, mapping of genes to module space gives information on the type of MPA signature exhibited by a gene, as well as the phenotypes involved in the signature. The high density of SNPs in this population and the rapid decay of LD allows for the high resolution of MPA signatures. Supplementary Figure 2A shows the variation in LD in the region including 5 kb upstream and downstream of Potri.001G419800, the type 2 MPA gene in Figure 10F. One can see that both associating variants in this gene are in a region of low LD. Supplementary Figure 2B shows a pairwise LD heatmap of 100 variants in this region including the two associating variants in Potri.001G419800. One can see that these two associating variants exist within two separate LD blocks.

The beta value derived from each SNP-phenotype association gives an indication of the effect that the SNP has on the value of the phenotype. One can look at the beta values from the GWAS analysis to see if the minor allele of a given SNP has statistically a positive or negative affect on the phenotype value. This will inform the researcher of the potential functional affect of each SNP. Overall, positive and negative beta values are present in associations in the set of type 1 MPA genes, type 2 MPA genes and single phenotype association (SPA) genes, although negative beta values are far more prevalent across all categories (Supplementary Figure 3) indicating that most minor alleles have negative effects on the phenotype (metabolite) values.

**Figure 11:**
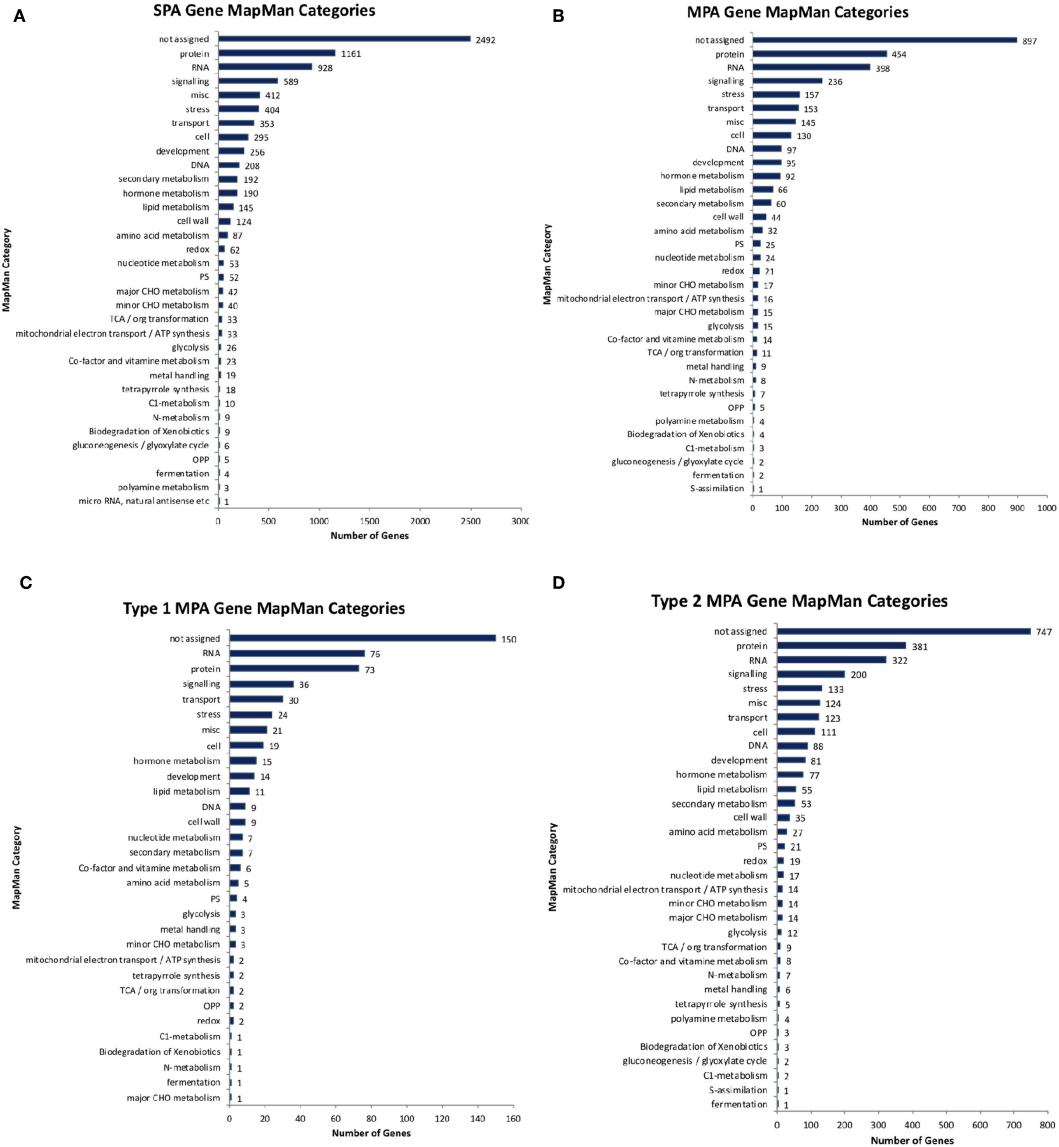
Functional annotations. Number of genes annotated with different high-level MapMan categories for **(A)** non-MPA genes, **(B)** all MPA genes, **(C)** type 1 MPA genes, and **(D)** type 2 MPA genes.

Of the 10,566 genes that had at least one phenotype hit, 2,964 exhibited a MPA signature by associating with more than one phenotype (Supplementary Figure 4A). Of those MPA genes, type 2 MPA signatures were far more abundant, with 2,468 genes exhibiting a type 2 MPA signature and 496 genes exhibiting a type 1 MPA signature (Supplementary Table 1, Supplementary Figure 4B). MPA genes represented a broad range of functions (Figure 11). No functional enrichment was found in the set of type 1 MPA genes. However, various GO terms were found to be enriched in the set of type 2 MPA genes, including developmental functions such as root development, shoot development, leaf development, fruit development, symbiosis, encompassing mutualism through parasitism, various regulatory functions such as RNA gene silencing function and response to stress and DNA repair (see Supplementary Figures 5–7, Supplementary Table 2, Supplementary File 1 for complete enrichment results).

Chaperones are classic examples of pleiotropic genes, assisting in the folding of various proteins. (Sung and Guy, 2003; Sangster et al., 2004; Gong and Golic, 2006). Querying the MPA networks for potential pleiotropic chaperones, we uncovered 14 potential chaperones based on there best *Arabidopsis* hit annotation, that contain MPA signatures (Supplementary Table 3), 12 of which contain type 2 MPA signatures. It is encouraging to see these classic pleiotropic genes appearing in the MPA networks, and interesting that they mostly exhibit type 2 MPA signatures.

### 3.3. Signature Clustering in Powerset Space

**Figure 12:**
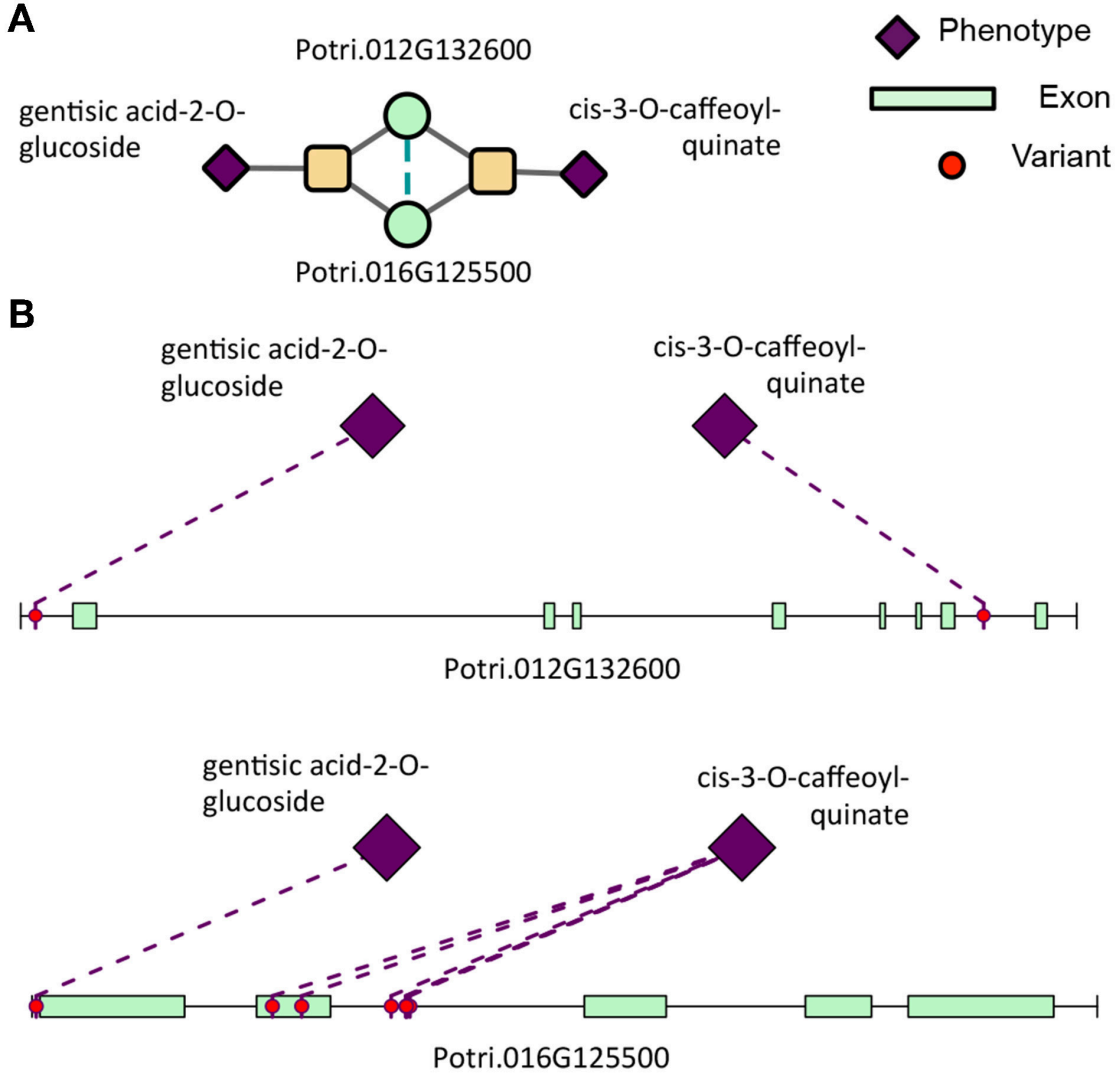
Type 2 signature cluster. **(A)** Signature cluster defined by a Type 2 association with gentisic acid-2-O-glucoside and cis-3-O-caffeoyl-quinate. **(B)** Associating SNP positions within genes in this signature cluster. These SNP associations have negative effect sizes (beta values) on the phenotype values. See Table 1 for gene information.

Clustering of genes in phenotype space produces groups of genes with the same overall set of phenotype associations. However, it does not provide any information as to the topology of Type 1/Type 2 associations of SNPs within the gene. Powerset space is defined by sets of phenotypes, and thus, clustering genes in this space groups genes based on the topology of Type 1/Type 2 associations of SNPs within the gene. After mapping genes to the newly constructed powerset space, genes were clustered (Figure 2F, *Methods and Materials*) resulting in groups of genes containing the same MPA signature. Members of a given cluster represented genes harboring identical MPA signatures. This means that genes within the same signature cluster have associations with the same modules. For example, the signature cluster driven by two modules, one involving associations with cis-3-O-caffeoyl-quinate and the other involving associations with gentisic acid-2-O-glucoside contains two genes, Potri.016G125500.v3.0 (homolog of *Arabidopsis thaliana* TRICHOME BIREFRINGENCE-LIKE 34) and Potri.012G132600.v3.0 (homolog of *Arabidopsis thaliana* AGAMOUS-like 6). These genes have associations with both cis-3-O-caffeoyl-quinate and gentisic acid-2-O-glucoside, however a given SNP within these genes is associated with either caffeoyl-quinate *or* gentisic acid-2-O-glucoside, but not both (Figure 12). This exemplifies what MPA decomposition and signature clustering accomplishes—the extraction of detailed multi-phenotype association signatures within genes, and the grouping of genes based on these detailed MPA signatures.

**Table 1:** IDs, *Arabidopsis thaliana* best hits and corresponding descriptions of genes in the gentisic acid/cis-3-caffeoyl-quinate signature cluster (Figure 12).

**Gene ID**	***A. thaliana* best hit**	**Description**
Potri.012G132600	AT2G45650	AGAMOUS-like 6
Potri.016G125500	AT2G38320	TRICHOME BIREFRINGENCE-LIKE 34

**Figure 13:**
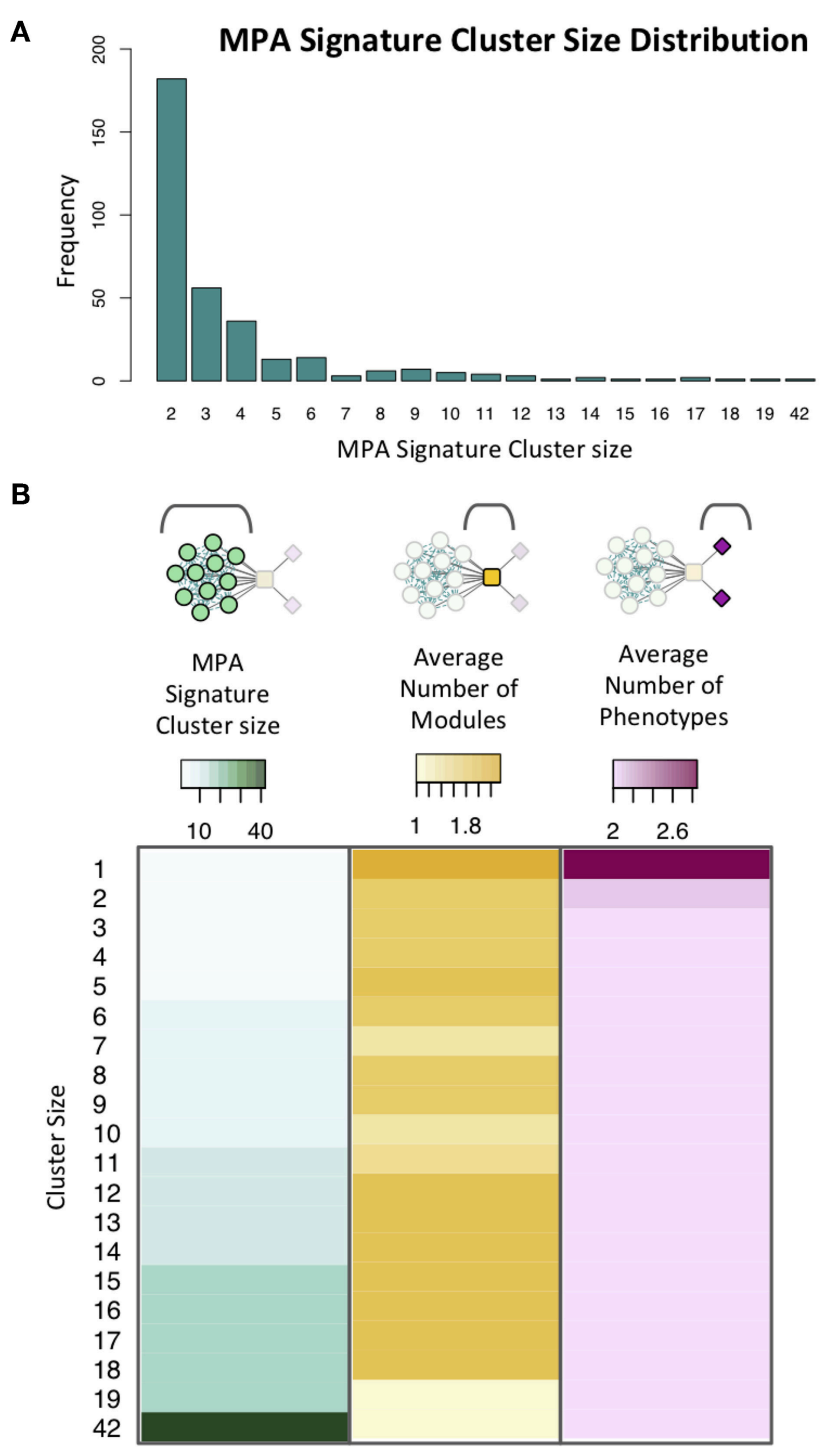
Signature clusters in powerset space. **(A)** Cluster size distribution for signature clusters containing ≥ 2 genes. **(B)** Heatmap showing cluster size (green), average number of modules associated with genes of a given cluster size (yellow) and average number of phenotypes associated with genes in clusters of a given size (pink).

MPA signature clusters varied in size and complexity, ranging from large sets of genes having simple MPA signatures (Supplementary Figures 8A,B; Supplementary Table 4) to single gene clusters harboring very complex MPA signatures (Supplementary Figures 8C,D). An inverse relationship existed between the cluster size, and the number of associated phenotypes, with a minimum gene cluster size of one and a maximum gene cluster size of 42 (Figure 13). Complex MPA signatures are possible in this population partly because of the rapid rate with which Linkage Disequilibrium (LD) decays, dropping below 0.2 within 100 bp (Supplementary Figure 9).

**Figure 14:**
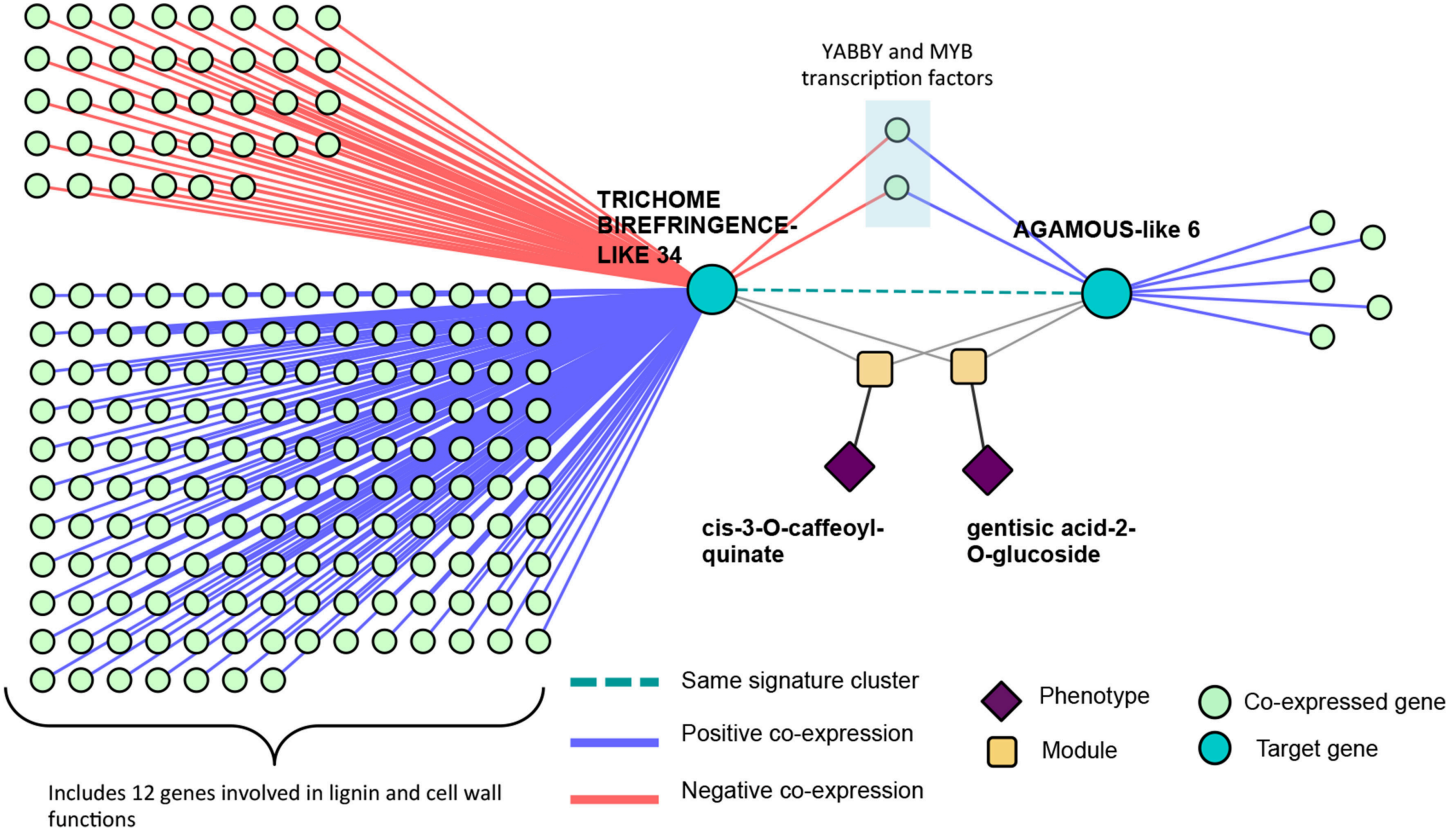
Co-expression lines of evidence. Co-expression relationships of the signature cluster consisting of TBL34 and AGL6 from Figure 12.

These signature clusters are easily combined with other data types in a “lines of evidence” fashion, as introduced in Weighill et al. (2018). Signature clusters such as those in Figure 12 can be merged with their neighbors in a co-expression network, providing additional insights into the functioning of these genes. Potri.016G125500 (TBL34) and Potri.012G132600 (AGL6) appeared in the same signature cluster, and are associated with many cell-wall related genes/phenotypes. TBL34 and AGL6 both associated with gentisic acid-2-O-glucoside and cis-3-O-caffeoyl-quinate, and both co-expressed with the same two transcription factors (Figure 14). An interesting regulatory circuit is potentially revealed, in that AGL6 potentially activates two transcription factors (positive co-expression edges) which, in turn potentially repress TBL34 (negative co-expression edges). TBL34 is also positively co-expressed with 12 genes involved in cell wall and lignin biosynthesis functions (Figure 14). TBL genes are known to o-acetylate xylose (Gille et al., 2011), a function which has been found to be essential for resistance to certain pathogens (Gao et al., 2017). Gentisic acid and its conjugate is a pathogen-induced signaling molecule (Bellés et al., 1999) which itself has been found to induce pathogen resistance in plants (Campos et al., 2014) and induce expression of pathogenesis-related proteins (Bellés et al., 1999). Various AGL genes are also cell-wall related in that they impact lignin content (Ferrándiz et al., 2000; Giménez et al., 2010; Cosio et al., 2017). This could be a regulatory circuit of biotic-stress-related cell wall remodeling, in which AGL6 potentially regulates xylose o-acetylation via TBL34.

### 3.4. Extensions to Pleiotropy

Several definitions of pleiotropy involve a gene associating with multiple, apparently disparate, unrelated phenotypes (see for example Stearns, 2010), and not all MPAs can be interpreted as pleiotropic signatures. However, if the two phenotypes are disparate enough, one can begin to hypothesize about potential pleiotropic functioning of the gene in question. In this particular study, we demonstrated our method on a collection of molecular phenotypes of metabolite concentrations. If two metabolites in a MPA exist within separate pathways, one could consider it a potentially pleiotropic interaction.

A particular example of this phenomenon found in our analysis is Potri.002G178400. This gene has a type 2 MPA association with shikimic acid and raffinose (Supplementary Figure 10). Based on existing knowledge found in PlantCyc on the Plant Metabolic Network (PMN) online resource (Schlapfer et al., 2017), these two metabolites are found in different pathways. Shikimic acid is involved in reactions in pathways “chlorogenic acid biosynthesis I,” “chlorogenic acid biosynthesis II,” “phaselate biosynthesis,” “phenylpropanoid biosynthesis,” “simple coumarins biosynthesis,” and “chorismate biosynthesis from 3-dehydroquinate” whereas raffinose is involved in reactions in pathways “lychnose and isolychnose biosynthesis,” “stellariose and mediose biosynthesis,” “ajugose biosynthesis II (galactinol-independent),” “stachyose degradation,” and “stachyose biosynthesis.” Supplementary File 2 contains a high resolution PDF showing the positions of raffinose (red boxes) and shikimic acid (blue box) in the *P. trichocarpa* Cellular Overview metabolic map generated on the Plant Metabolic Network online resource. Potri.002G178400 contains two Pfam domains, namely pfam01565 (FAD binding domain) and pfam04030 (D-arabinono-1,4-lactone oxidase). This is an interesting example of a potentially pleiotropic gene, which affects two different metabolic phenotypes. A possible explanation for the mechanism of this pleiotropic interaction is through competition for carbon, with shikimic acid committing carbon to secondary metabolism and raffinose being the product of storage for primary carbon metabolism.

It is however important to note that pleiotropic signatures can be difficult to disentangle true pleiotropic associations from other multi-phenotype associations, and should be addressed carefully. Multi-phenotype associations can be interpreted as true pleiotropy, but could also be various forms of spurious pleiotropy (see Solovieff et al., 2013 for a useful review).

### 3.5. Future Prospects and Implications

*P. trichocarpa* was an ideal species for the demonstration of the MPA decomposition for several reasons. Firstly, a large collection of 1,100 *P. trichocarpa* accessions have been clonally propagated in common gardens, resequenced and genotyped, (Tuskan et al., 2006; Slavov et al., 2012; Evans et al., 2014) providing a dense set of ~28 million variants which are publicly available (DOI 10.13139/OLCF/1411410). Secondly, linkage disequilibrium (LD) decays very rapidly within this population of *P. trichocarpa* (Supplementary Figure 9). This, in combination with the dense SNP genotyping, allowed for very fine-scale MPA signatures to be resolved. Thirdly, many other different 'omics datasets exist for *P. trichocarpa* including genome scale methylation data across 10 different tissues (Vining et al., 2012) as well as a gene expression atlas are available on Phytozome (Goodstein et al., 2012). This provides extra data layers which can be integrated with the MPA networks in order to provide further interpretation and context to the GWAS associations seen in the MPA signatures, in a Lines of Evidence approach (Weighill et al., 2018). Lastly, Poplar is an important bioenergy crop (Sannigrahi et al., 2010) and is the target of extensive research. Thus, this method should be highly valuable to researchers aiming to attempt to genetically modify *P. trichocarpa* in order to impact phenotypes important to bioenergy.

The ease with which these MPA networks can be integrated with other network layers such as co-expression, co-methylation and SNP co-evolution networks provides a powerful strategy for furthering understanding and knowledge about the components of the system, which could aid in the annotation of genes/metabolites of previously unknown function.

Other previously published methods are able to provide information on multi-phenotype associations. The MARV (Multi-phenotype Analysis of Rare Variants) method (Kaakinen et al., 2017a) is a rare variant test that associate a gene with single or multiple phenotypes, with rare variants collapsed, so the result is gene-to-phenotype or gene-to-multi-phenotype association. This is a very valuable method to determine the potential multi-phenotype associations of a gene harboring rare variants. This method however results in a score for each gene indicating its association with a set of phenotypes, and SNP-phenotype associations within the gene are not reported. Cichonska et al. (2016) present a method of performing SNP-to-multi-phenotype and multi-SNP-multi-phenotype associations. Another method by Mägi et al. (2017) associates SNPs with multiple phenotypes through a “reverse regression” approach, using phenotypes as the predictors in the model. Both of these methods can provide a unified measure of a given variant's association with multiple phenotypes, and thus could prove to be a valuable alternative to standard univariate GWAS approaches and potentially provide an alternative, useful input set of SNP-multi-phenotype input associations to be characterized and clustered using MPA decomposition.

MPA decomposition produces signature clusters from GWAS results which can easily be merged with other data types for further interpretation. It is intended that this method will be a valuable tool in the planning of future genetic modification experiments. The resolution of the MPA signatures revealed by this method provides a useful tool to use alongside new CRISPR-based gene editing technologies to achieve high precision genome editing. This method thus provides an informed strategy for increasing the precision of future synthetic biology efforts. Researchers aiming to modify a specific gene in order to impact a particular phenotype can select genes from the signature cluster best suited to the functions they want to modify. The module decomposition also provides information as to which variants/parts of genes are associating with one phenotype or more than one phenotype, and thus can inform the researcher whether the modification of a particular location within a gene will affect more than one phenotype.

MPA decomposition will also be particularly useful in the processing and interpretation of large GWAS datasets such as eQTN studies, involving associations between millions of variants and tens of thousands of phenotypes. Future application of this method to the expanding pool of phenotypic data available will allow for the generation of comprehensive signature clusters representing the global pleiotropic potential of a given organism, and inform the planning and precision of future synthetic biology efforts to impact a wide variety and scale of phenotypes. As such, this approach should have broad impacts by developing high resolution models of MPA/pleiotropy prediction that will form the foundation of future bioengineering design efforts.

## Author Contributions

DJ conceived of the study and supervised the project. DW developed MPA decomposition and signature clustering, implemented the method, generated and interpreted results and wrote the manuscript. PJ and CB performed the GWAS and outlier analysis. PR, NZ, MM, and TT performed the metabolomics. JS and AS contributed the genome sequence and transcriptome expression analysis. MS mapped gene expression atlas reads and calculated gene expression TPM values. GT led the sequencing of *Populus* genotypes. SD and DM-S performed the SNP calling and validation. DJ, GT, TT, PJ, DM-S, and SD provided editorial feedback on the manuscript.

### Conflict of Interest Statement

The authors declare that the research was conducted in the absence of any commercial or financial relationships that could be construed as a potential conflict of interest.
