# Supplementary material for: Multi-Phenotype Association Decomposition: Unraveling Complex Gene-Phenotype Relationships
Source: Front Genet. 2019 May 10;10:417. doi: 10.3389/fgene.2019.00417 (PMC6522845; doi:10.3389/fgene.2019.00417)
Supplement: Supplementary file 2 [file Data_Sheet_1.pdf]

Supplementary Material: Multi-Phenotype Association Decomposition: Unraveling complex gene-phenotype relationships

SUPPLEMENTARY TEXT

Text S1: Proportional Similarity Threshold

A proportional similarity threshold of 1 was chosen when calculating the similarity between the SNP vectors in phenotype space. While this might seem overly stringent, we particularly want to extract groups of SNPs with *identical* phenotype associations in order to form the modules. This is what allows us to easily define modules as equivalence classes. Otherwise, our modules would not represent elements of the powerset of phenotypes observed in SNP-phenotype associations. These modules (elements of the powerset of phenotypes) allow us to rigorously and precisely characterize the MPA signature of genes, which subsequently allows us to cluster genes based on their MPA signatures. For other purposes, for example, if one wants to simply cluster SNPs/genes to obtain groups of genes with similar phenotype associations for functional analysis, one could adjust this threshold. However, for our purposes, in order to characterize *exact* MPA signatures to aid in the planning of genetic modification experiments, we chose a threshold of 1.

Text S2: Formal Definition of Association Modules

Let the SNP-phenotype GWAS network G be defined as $G = (U, V, E)$, where U is the set of all SNPs within genes with at least two phenotype associations, V is the set of all phenotypes with at least one SNP association and E is the set of edges defined as:

$$E = \{\{u, v\} | u \in U \wedge v \in V \wedge u \text{ is significantly associated with } v\} \tag{S1}$$

We will define association modules as equivalence classes of U under the relation R. Notation as in MacLane and Birkhoff (1988) will be used.

First we define the binary relation R for any $x, y \in U$ as:

$$xRy \iff PS(x, y) = 1 \tag{S2}$$

where $PS(x, y)$ is the Proportional Similarity between x and y (see *Methods and Materials*). Since it is true that:

$$PS(x, x) = 1 \tag{S3}$$

$$PS(x, y) = PS(y, x) \tag{S4}$$

$$PS(x, y) = 1 \wedge PS(y, z) = 1 \implies PS(x, z) = 1 \tag{S5}$$

we have that reflexivity, symmetry and transitivity hold:

$$xRx \text{ is true} \tag{S6}$$

$$xRy \iff yRx \tag{S7}$$

$$xRy \wedge yRz \implies xRz \tag{S8}$$

Thus we have that R is an equivalence relation. We define the equivalence class of any element $x \in U$ as:

$$P_R x = \{y | y \in U \wedge yRx\} \tag{S9}$$

Each association module M_i is defined as an equivalence class of U under the relation R.

Text S3: Formal Definitions of MPA Matrices

Below we provide mathematical definitions for the construction of the MPA matrices.

Recall that the SNP-phenotype network G is defined as $G = (U, V, E)$, where U is the set of all SNPs with at least one phenotype hit, V is the set of all phenotypes with at least one SNP hit and E is the set of edges defined as:

$$E = \{\{u, v\} | u \in U \wedge v \in V \wedge u \text{ is significantly associated with } v\} \tag{S10}$$

We define S_{G_i} to represent the set of SNPs which reside within gene G_i. The gene-phenotype matrix GP is constructed such that each row $G_i \in \{G_1...G_m\}$ represents a gene, and each column $P_i \in \{P_1...P_l\}$ represents a phenotype. We define each entry GP_{ij} of the gene-phenotype matrix as:

$$GP_{ij} = \begin{cases} 1 & \text{if } \exists s \in U | s \in S_{G_i} \wedge \{s, P_j\} \in E \\ 0 & \text{otherwise} \end{cases} \tag{S11}$$

Intuitively, this means that entry GP_{ij} will be 1 if there exists a SNP within a MPA gene G_i that is associated with phenotype P_j, and 0 otherwise.

The gene-module matrix GM is constructed such that each row $G_i \in \{G_1...G_m\}$ represents a gene and each column $M_i \in \{M_1...M_n\}$ represents a association module. Each entry GM_{ij} is then defined as:

$$GM_{ij} = \begin{cases} 1 & \text{if } \exists s \in U | s \in S_{G_i} \wedge s \in M_j \\ 0 & \text{otherwise} \end{cases} \tag{S12}$$

Intuitively, this means that entry GM_{ij} will be a 1 if module M_j contains a SNP that resides within gene G_i, and zero otherwise.

The module-phenotype matrix MP was constructed such that each row $M_i \in \{M_1...M_n\}$ represents a association module, and each column $P_i \in \{P_1...P_l\}$ represents a phenotype. We define Q_{M_i} to be the set of phenotypes driving the correlation between the SNPs within module M_i, i.e:

$$Q_{M_i} = \{P_i \in V | \forall s \in M_i, \{s, P_i\} \in E\} \tag{S13}$$

We then define each entry of the module-phenotype matrix MP_{ij} to be:

$$MP_{ij} = \begin{cases} 1 & \text{if } P_j \in Q_{M_i} \\ 0 & \text{otherwise} \end{cases} \tag{S14}$$

We refer to the gene-module and module-phenotype matrices as the *decomposition matrices*, and refer collectively to the set of all three matrices (gene-phenotype GP, gene-module GM and module-phenotype MP matrices) as the *MPA matrices*.

Text S4: MPA Cube

The three MPA matrices can be seen as different sides of a *MPA cube* C as shown in Figure S1A. We define the first dimension of the cube to be genes, the second dimension to be association modules, and the third dimension to be phenotypes. We define each entry C_{ijk} to be:

$$C_{ijk} = \begin{cases} 1 & \text{if } (\exists s \in M_j | s \in S_{G_i}) \wedge (\forall s \in M_j : \{s, P_k\} \in E) \\ 0 & \text{otherwise} \end{cases} \tag{S15}$$

One can retrieve the individual MPA matrices from the MPA cube simply by "viewing" the cube from different angles, as illustrated in Figure S1B. Imagine a transparent box in the dimensions of the MPA cube being filled by $1 \times 1 \times 1$ small cubes. Each small cube is colored black if the corresponding entry in the MPA cube is 1, and transparent if the corresponding entry in the MPA cube is 0. Viewing the transparent box from different sides will reveal a pattern of black and transparent squares, representing the binary values in one of the three MPA matrices, depending on which side you are viewing the cube from. For example, in Figure S1B, viewing the cube from the top will reveal the MP matrix, while viewing the cube from the front will reveal the GP matrix and viewing the cube's right side will reveal the GM matrix.

Text S5: Composition and Decomposition Relationships

The three MPA matrices satisfy the following equation:

$$GP = \text{bin}(GM \cdot MP) \tag{S16}$$

where bin() is a binarizing function, setting all entries in a matrix which are greater than one to the value one, and $\cdot$ is normal matrix multiplication. This is a decomposition-like relationship, in that the GP matrix is, with the exception of the binarizing function, decomposed (or factorized) into matrices with an intervening latent variable, namely the association module variable.

The bipartite MPA networks can be seen to have a composition relationship as outlined in Figure 6 in the main text. If a gene G_i and a module M_j are connected in the GM network, and that module M_j is connected to phenotype P_k in the MP network, then gene G_i will be connected to phenotype P_k in the GP network.

SUPPLEMENTARY FILES

File S1. Cytoscape session: Cytoscape session containing interactive networks and p-values for the BINGO (Maere et al., 2005) results for Type 2 MPA genes.

File S2. Metabolic pathway: Positions of raffinose (red) and shikimate (blue) in the PlantCyc metabolic pathway map for *P. trichocarpa* on the Plant Metabolic Network (PMN) online resource (Schlapfer et al., 2017).

SUPPLEMENTARY TABLES

Table S1. MFA Genes: Gene IDs, SNP IDs, beta values and annotation information for MFA genes. Annotation information was derived from the version 3 genome annotation on Phytozome (Goodstein et al., 2012).

Table S2. GO terms: GO-terms and their associated adjusted p-values from the GO enrichment analysis, sorted by adjusted p-value. Interactive networks of the GO enrichment analysis per GO hierarchy (Biological Process, Molecular Function and Cellular Component) as well as the associated p-values can be found in the Cytoscape session in Supplementary File 1.

Table S3. Chaperones: Annotation information for the primary transcripts of the 14 chaperone-related genes identified as MPA genes. Functional information shown was obtained from the version 3.0 gene annotation of *P. trichocarpa* on Phytozome Goodstein et al. (2012) and includes PFAM domains, as well as the ID, name and description of the best *Arabidopsis thaliana* hit. The type of MPA signature exhibited (type 1 or type 2) is also shown.

Table S4: Gene annotation for Supplementary Figure S8: IDs, *Arabidopsis thaliana* best hits and corresponding descriptions of genes in the fatty acid signature cluster (Figure S8).

Gene ID	*A. thaliana* Best Hit	Description
Potri.003G122900	AT1G63120	RHOMBOID-like 2
Potri.006G188500	AT4G31985	Ribosomal protein L39 family protein
Potri.008G179800	AT3G26000	Ribonuclease inhibitor
Potri.014G117800	AT2G47230	DOMAIN OF UNKNOWN FUNCTION 724 6
Potri.019G074600	AT4G10030	alpha/beta-Hydrolases superfamily protein
Potri.019G074700	AT1G71490	Tetratricopeptide repeat (TPR)-like superfamily protein
Potri.019G075000	AT3G44540	fatty acid reductase 4
Potri.019G075200	AT3G44540	fatty acid reductase 4
Potri.019G075300	AT4G33790, AT3G44540	fatty acid reductase 4, Jojoba acyl CoA reductase-related male sterility protein
Potri.019G075400	AT1G71460	Pentatricopeptide repeat (PPR-like) superfamily protein
Potri.019G087100	AT4G12600	Ribosomal protein L7Ae/L30e/S12e/Gadd45 family protein

SUPPLEMENTARY FIGURES

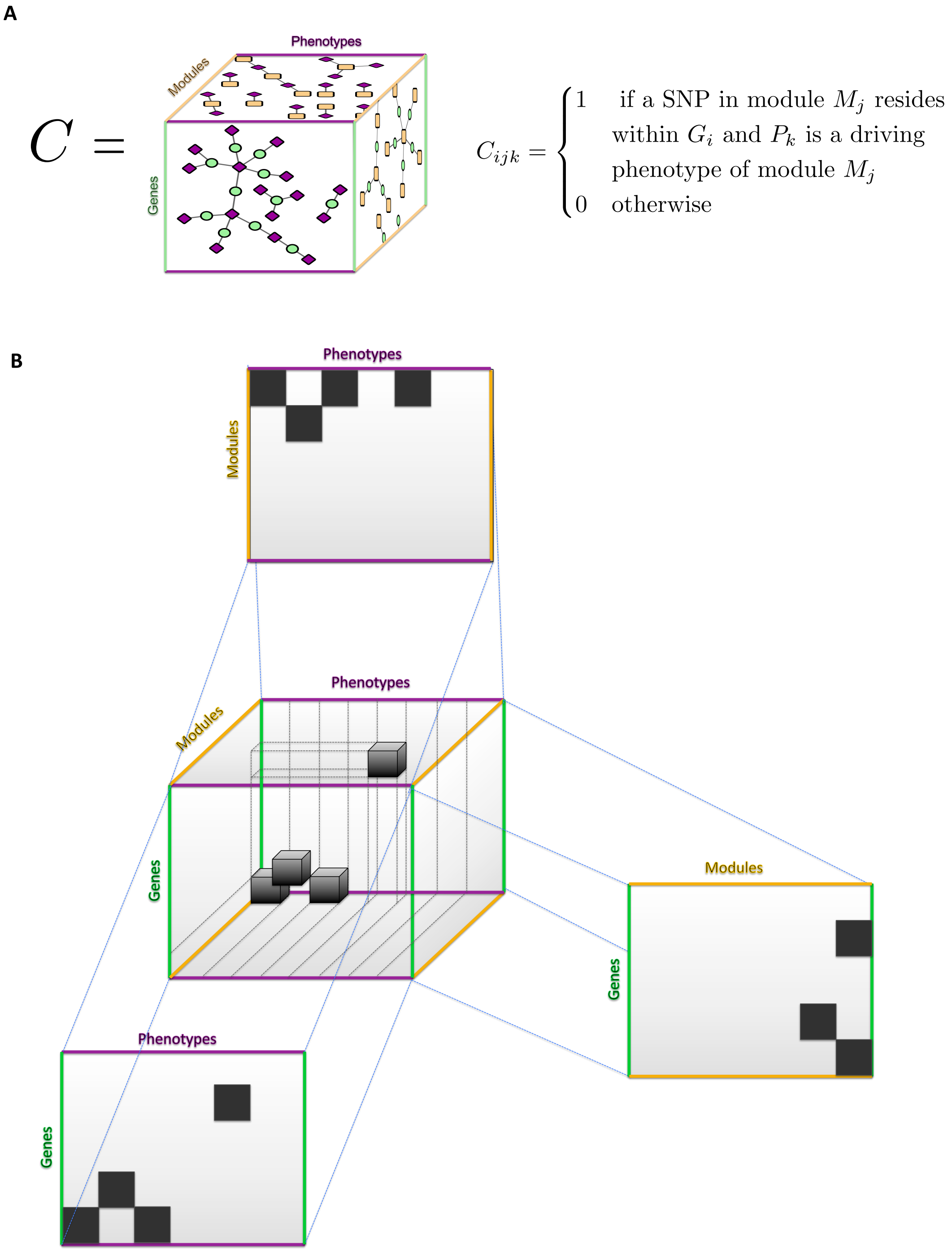

Figure S1. MPA Cube. (a) Definition of the MPA cube. (b) Projection onto a particular side of the cube results in one of the MPA matrices.

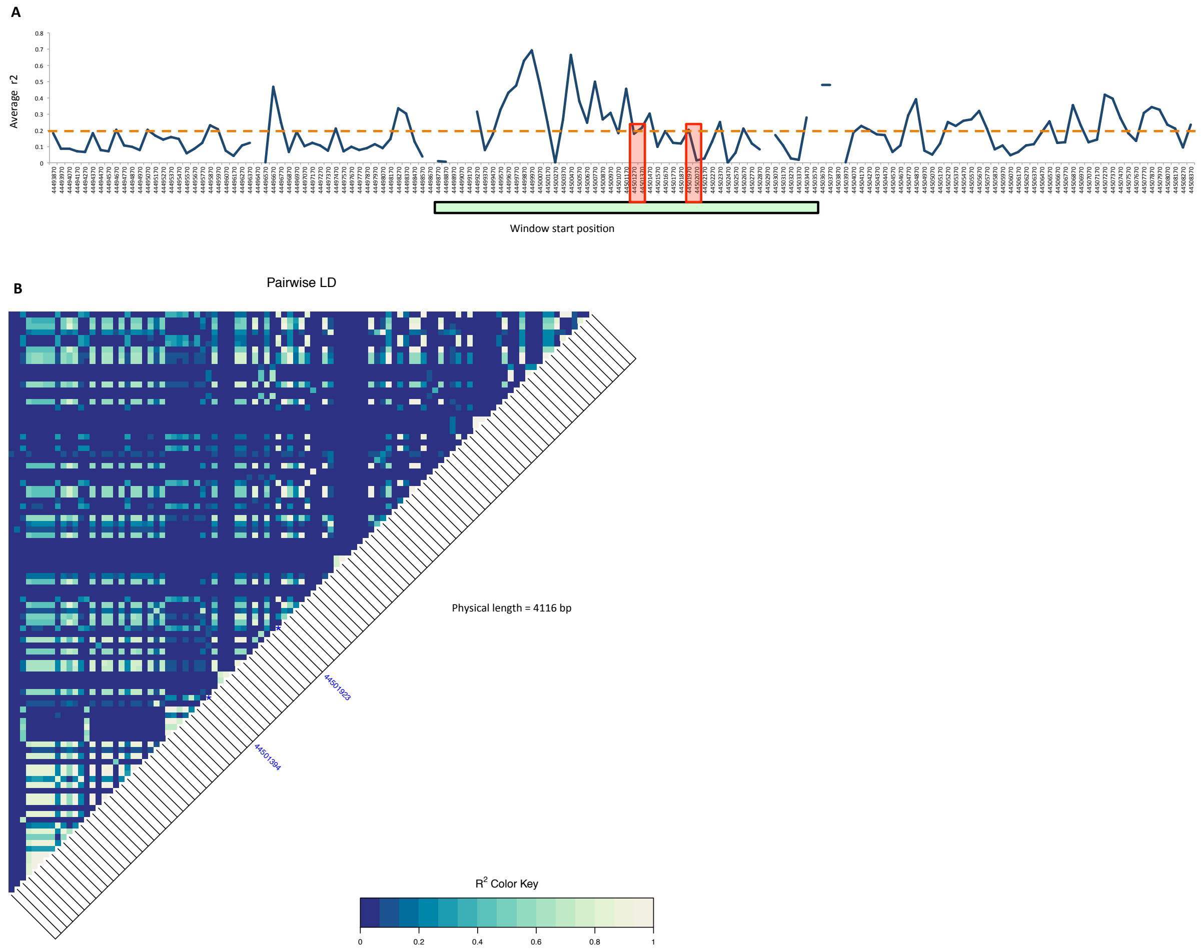

Figure S2. Local LD example. (A) Variation in LD in the region including 5kb upstream and downstream of Potri.001G419800. The green bar denotes the gene region, and then red bars highlight the overlapping bins containing the associating variants within the gene. LD r^2 values were calculated for pairs SNPs within 200bp windows across this region, overlapping by 100bp using PLINK (Purcell et al., 2007). (B) Pairwise LD heatmap of 100 variants in this region shown in (A) including the two associating variants in Potri.001G419800. LD values were calculated using PLINK (Purcell et al., 2007) and plotted using LDheatmap (Shin et al., 2006).

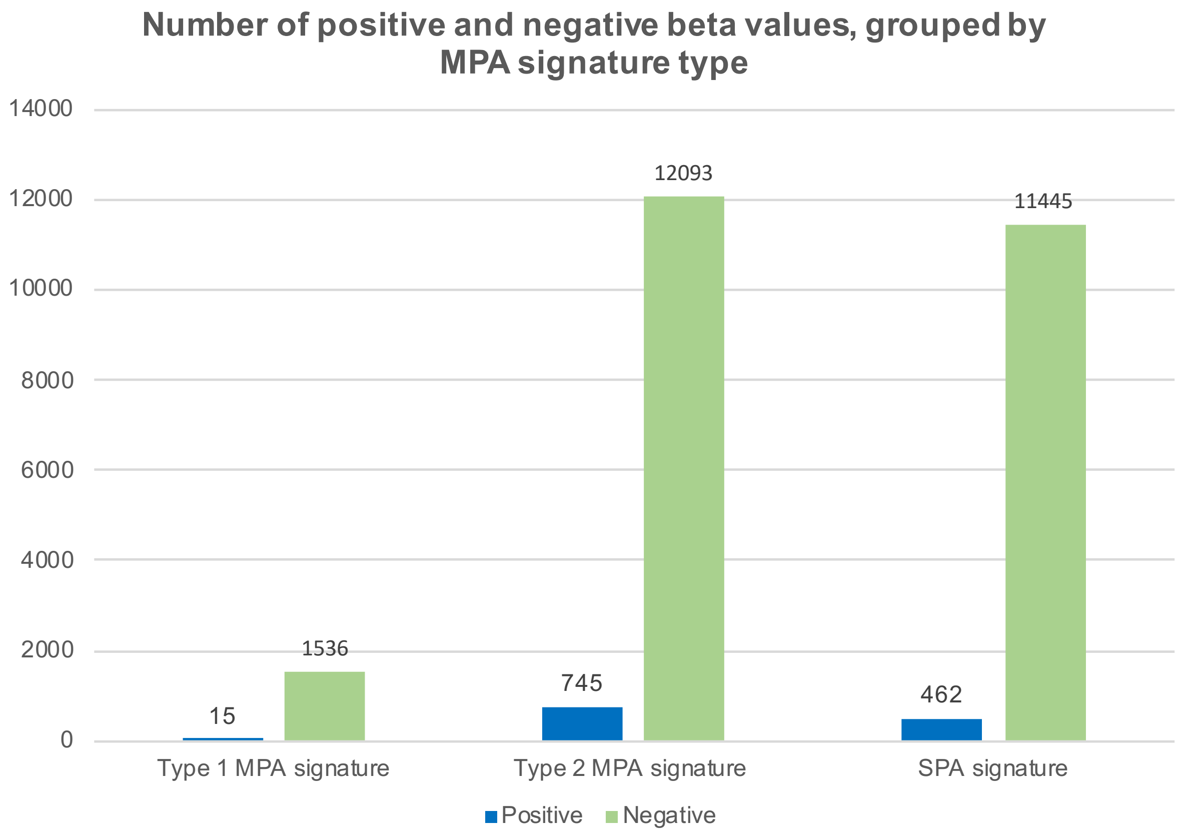

Figure S3. Beta values. Number of positive and negative significant beta values by MPA signature type.

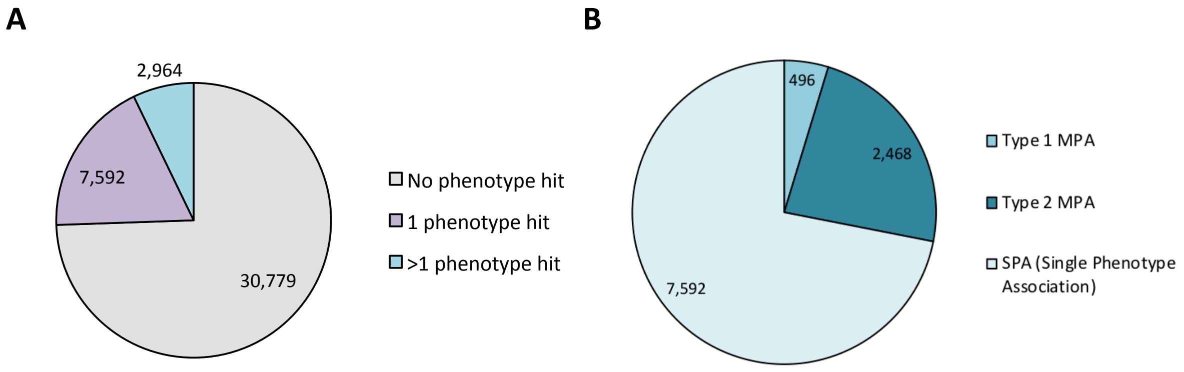

Figure S4. Gene GWAS Association Counts. Summary of the number of genes (A) with different numbers of GWAS phenotype hits and (B) different MPA signatures.

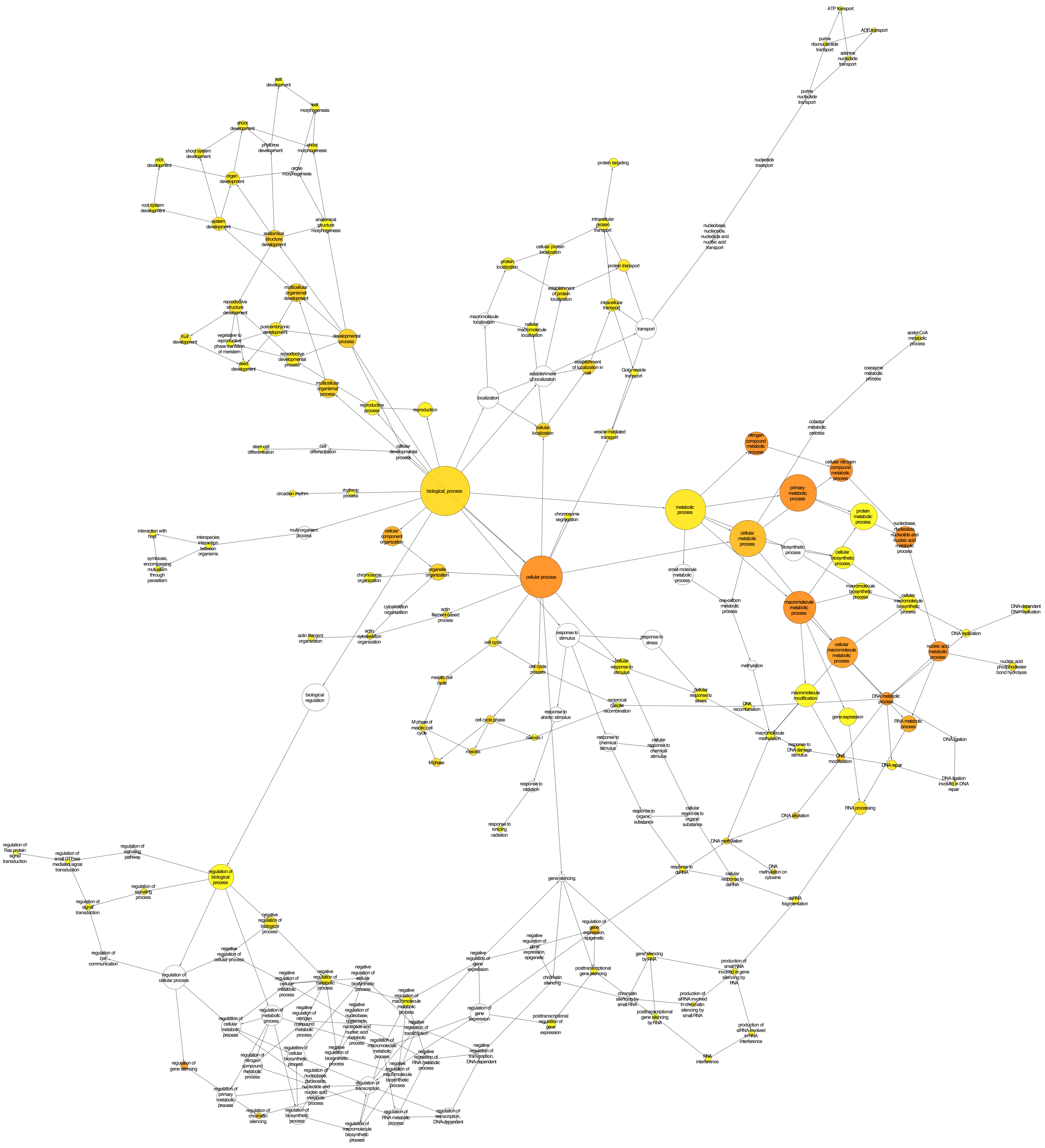

Figure S5. Biological Process Enrichment. Biological process GO terms enriched in the set of type 2 MPA genes. Enrichment was calculated using the BINGO Cytoscape plugin (Maere et al., 2005). Yellow/orange nodes represent significantly over-represented GO terms. The more intense the orange color, the more significant the p-value. White nodes represent GO terms that are not significantly over-represented, but are parents of over-represented terms in the GO hierarchy. Node size corresponds to the number of genes in that particular category in the set tested for enrichment. Interactive networks can be seen and zoomed in the Cytoscape (Shannon et al., 2003) session in Supplementary File 1.

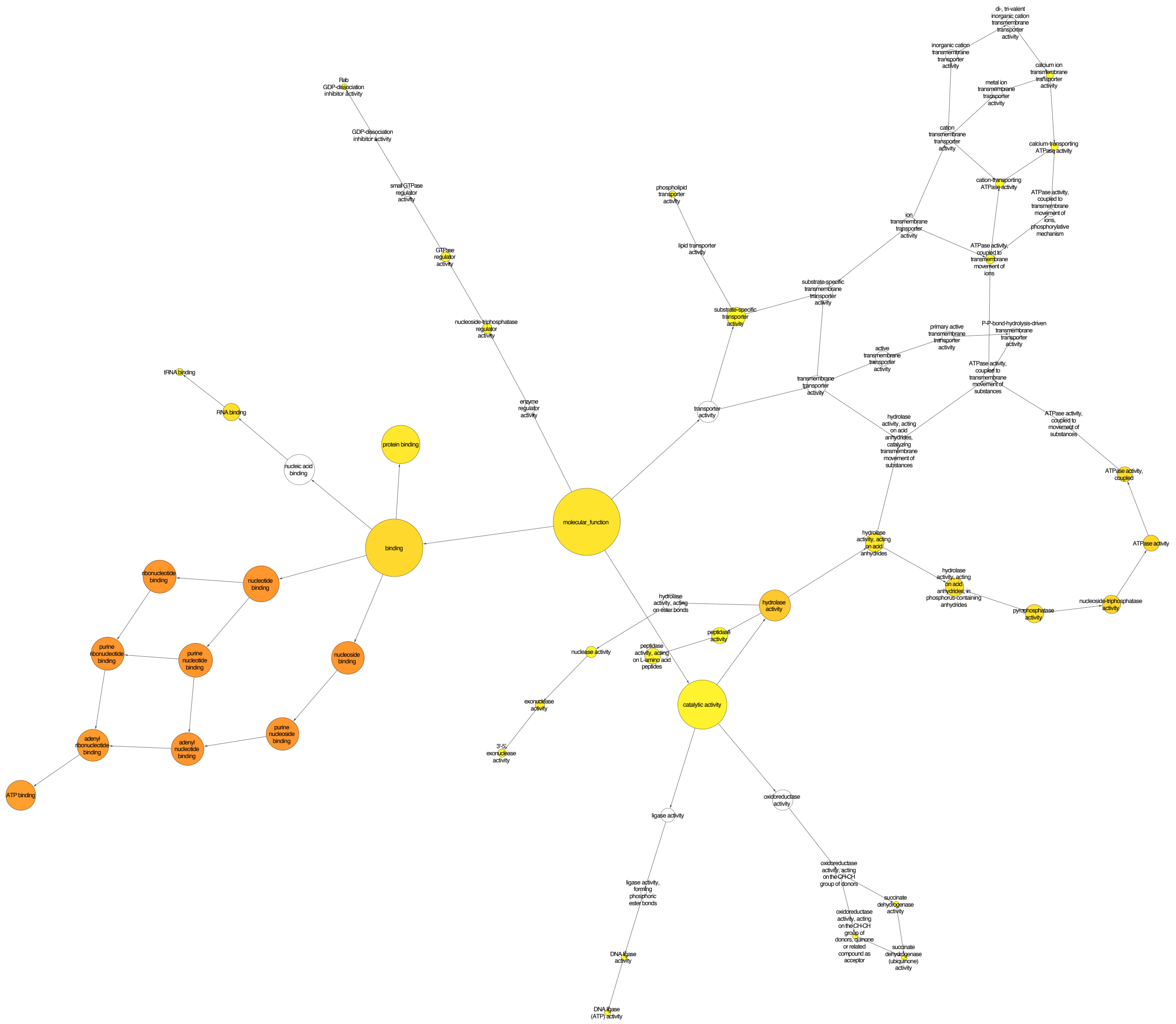

Figure S6. Molecular Function Enrichment. Molecular function GO terms enriched in the set of type 2 MPA genes. Enrichment was calculated using the BINGO Cytoscape plugin (Maere et al., 2005). Yellow/orange nodes represent significantly over-represented GO terms. The more intense the orange color, the more significant the p-value. White nodes represent GO terms that are not significantly over-represented, but are parents of over-represented terms in the GO hierarchy. Node size corresponds to the number of genes in that particular category in the set tested for enrichment. Interactive networks can be seen and zoomed in the Cytoscape (Shannon et al., 2003) session in Supplementary File 1.

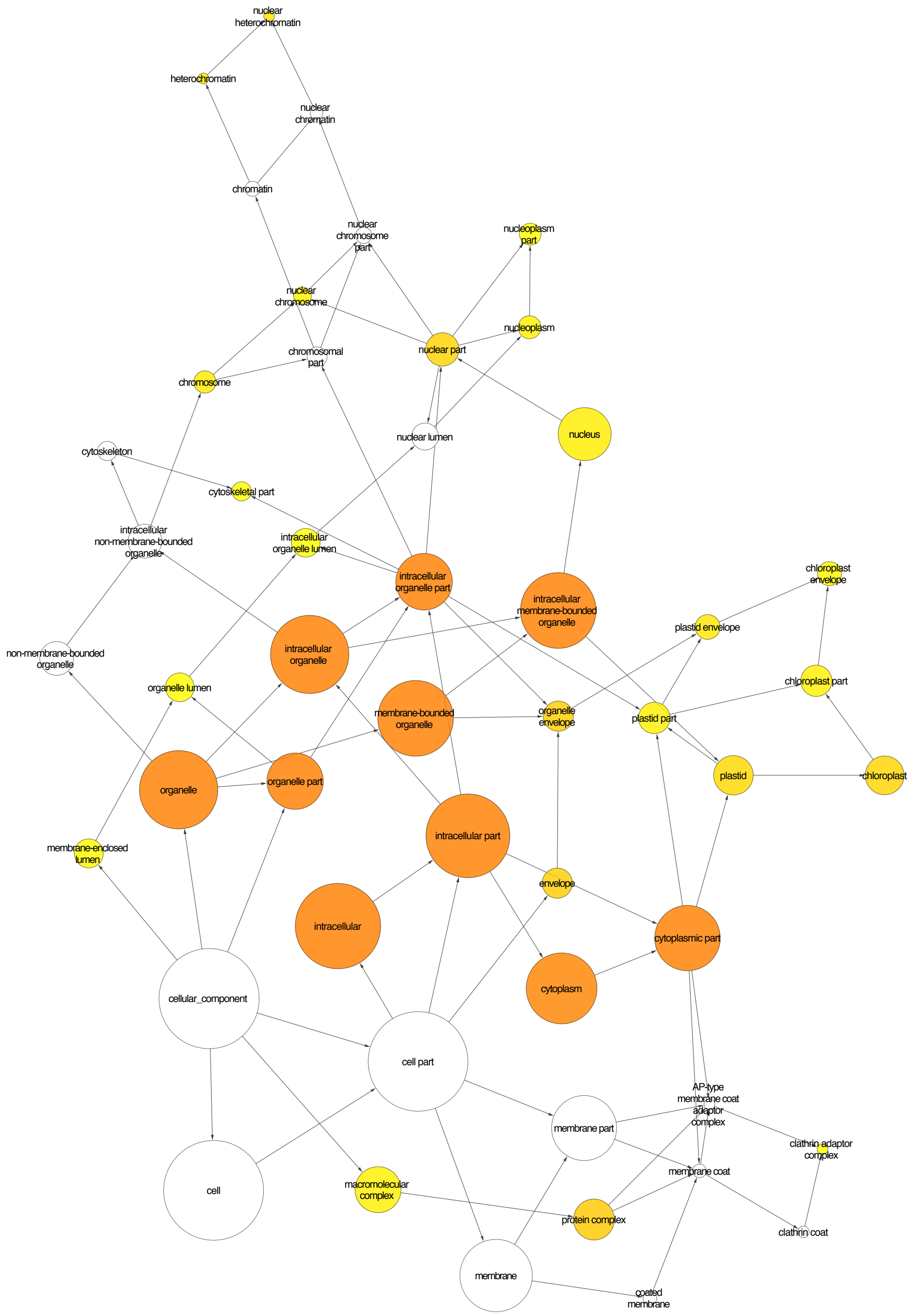

Figure S7. Cellular Component Enrichment. Cellular component GO terms enriched in the set of type 2 MPA genes. Enrichment was calculated using the BINGO Cytoscape plugin (Maere et al., 2005). Yellow/orange nodes represent significantly over-represented GO terms. The more intense the orange color, the more significant the p-value. White nodes represent GO terms that are not significantly over-represented, but are parents of over-represented terms in the GO hierarchy. Node size corresponds to the number of genes in that particular category in the set tested for enrichment. Interactive networks can be seen and zoomed in the Cytoscape (Shannon et al., 2003) session in Supplementary File 1.

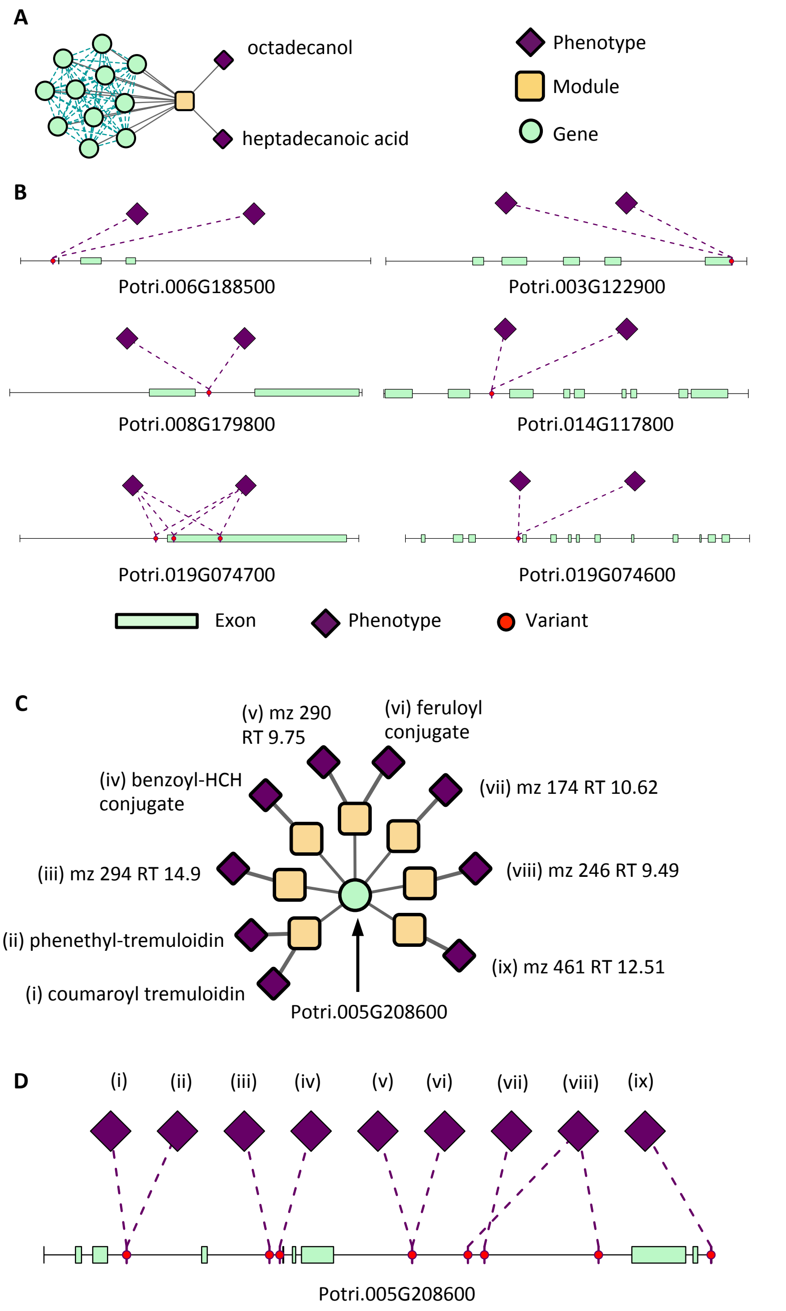

Figure S8. Simple and Complex MPA Signatures. (A) Signature cluster defined by a type one SNP association with octadecanol and heptadecanoic acid. See Supplementary Table S4 for gene information. (B) Associating SNP positions within a selection of the genes in this signature cluster. These SNP associations have negative effect sizes (beta values) on the phenotype values. (C) Single-gene cluster of Potri.005G208600, bearing a unique, complex MPA signature consisting of 7 modules and 9 phenotypes. (D) Associating SNP positions of Potri.005G208600. These SNP associations have negative effect sizes (beta values) on the phenotype values.

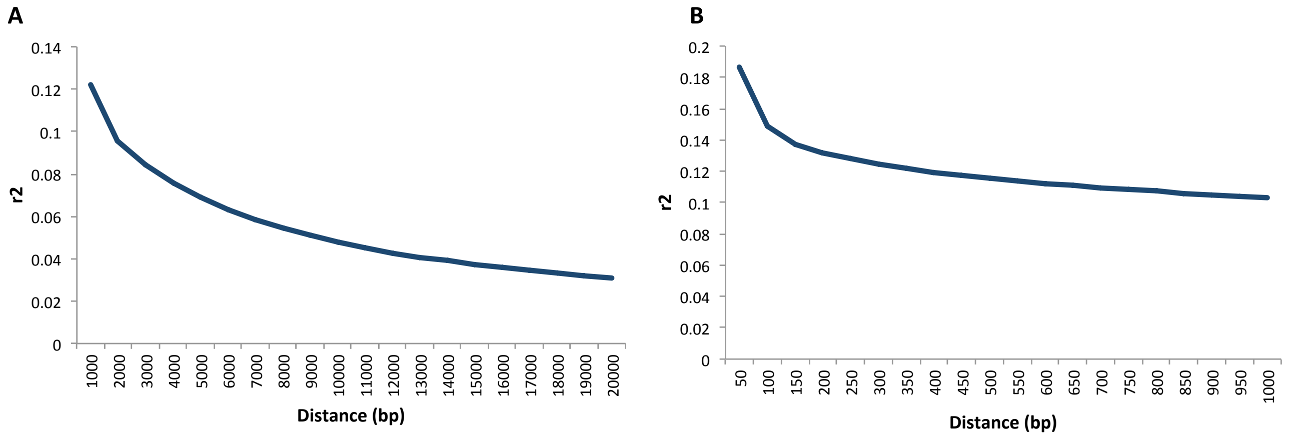

Figure S9. Decay of Linkage Disequilibrium. The decay of LD r^2 values plotted as the average r^2 value (y-axis) for SNPs within a given distance from each other (x-axis), for a length of (A) 20kb in 1kb windows and (B) 1kb in 50bp windows. LD values were calculated using PLINK (Purcell et al., 2007).

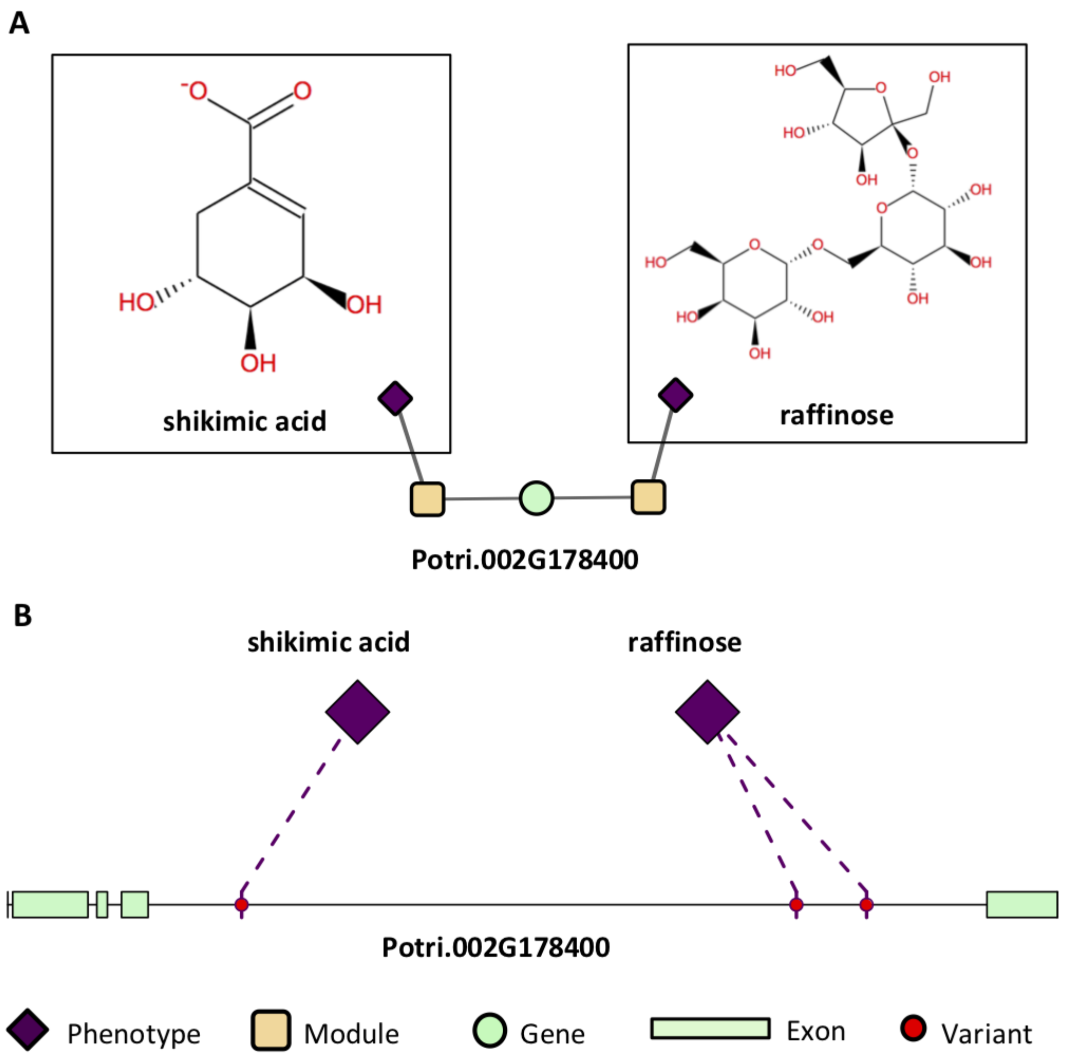

Figure S10. Pleiotropic Signature. (A) An example of a potentially pleiotropic signature of Potri.002G178400, involving a type 2 MPA with two metabolites in different pathways. (B) Associating SNP positions within Potri.002G178400.

REFERENCES

Goodstein, D. M., Shu, S., Howson, R., Neupane, R., Hayes, R. D., Fazo, J., et al. (2012). Phytozome: a comparative platform for green plant genomics. *Nucleic Acids Research* 40, D1178–D1186

MacLane, S. and Birkhoff, G. (1988). *Algebra* (AMS Chelsea Publishing), third edn.

Maere, S., Heymans, K., and Kuiper, M. (2005). Bingo: a cytoscape plugin to assess overrepresentation of gene ontology categories in biological networks. *Bioinformatics* 21, 3448–3449

Purcell, S., Neale, B., Todd-Brown, K., Thomas, L., Ferreira, M. A., Bender, D., et al. (2007). Plink: a tool set for whole-genome association and population-based linkage analyses. *The American Journal of Human Genetics* 81, 559–575

Schlapfer, P., Zhang, P., Wang, C., Kim, T., Banf, M., Chae, L., et al. (2017). Genome-wide prediction of metabolic enzymes, pathways and gene clusters in plants. *Plant physiology* , pp–01942

Shannon, P., Markiel, A., Ozier, O., Baliga, N. S., Wang, J. T., Ramage, D., et al. (2003). Cytoscape: A Software Environment for Integrated Models of Biomolecular Interaction Networks. *Genome Research* 13, 2498–2504

Shin, J.-H., Blay, S., McNeney, B., and Graham, J. (2006). Ldheatmap: An r function for graphical display of pairwise linkage disequilibria between single nucleotide polymorphisms. *J Stat Soft* 16, Code Snippet 3
