# Supplementary figures and images for: Multi-Phenotype Association Decomposition: Unraveling Complex Gene-Phenotype Relationships
Source: Front Genet. 2019 May 10;10:417. doi: 10.3389/fgene.2019.00417 (PMC6522845; doi:10.3389/fgene.2019.00417)

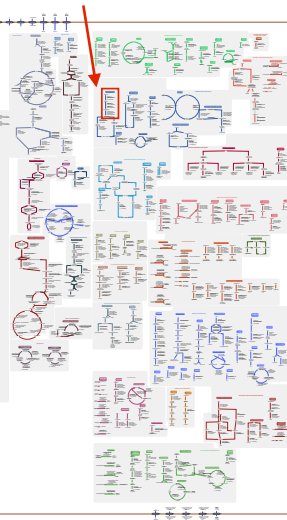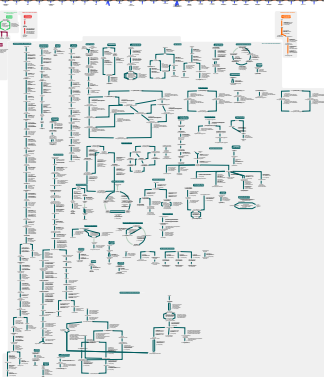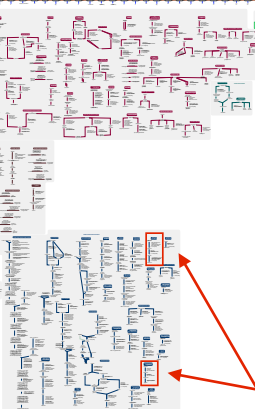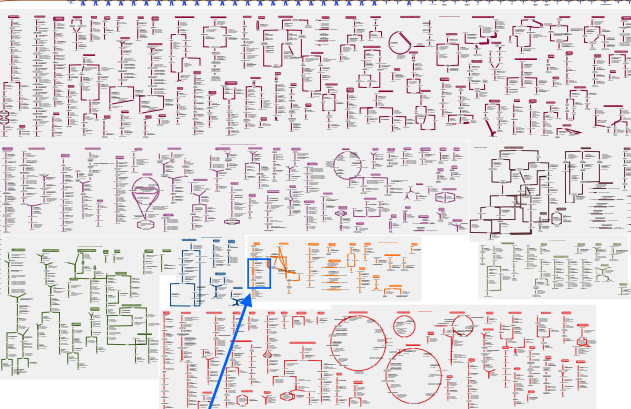

Supplement: Supplementary file 4 [file Data_Sheet_3.PDF]
